# Nanoparticles Against Antimicrobial Resistance: Chemical Design, Assembly, and Emerging Applications

**DOI:** 10.1021/acsnano.6c09306

**Published:** 2026-09-16

**Authors:** Thomas Daniel Vogelaar, Håvard Jenssen, Reidar Lund

**Affiliations:** † Department of Chemistry, University of Oslo, Postboks 1033 Blindern, 0315 Oslo, Norway; ‡ Department of Science and Environment, 6976 Roskilde University, Universitetsvej 1, 4000 Roskilde, Denmark; § Hylleraas Centre for Quantum Molecular Sciences, University of Oslo, Postboks 1033 Blindern, 0315 Oslo, Norway; ∥ Donostia International Physics Centre (DIPC), Manuel Lardizabal Ibilbidea, 4, 20018 Donostia/San Sebastián, Gipuzkoa, Spain

**Keywords:** antibiotics, antimicrobial resistance, biofilms, coatings, nanoparticles, nanotherapeutics, polymer-based, soft matter, supramolecular assembly, translational medicine

## Abstract

Antimicrobial resistance (AMR) is an escalating global health concern, with increasing rates of morbidity and mortality associated with multidrug-resistant infections worldwide. Despite intensive efforts to discover antimicrobial compounds, few candidates progress to clinical trials, and even fewer achieve market approval. A promising approach to address the current stagnation in antibiotic discovery is the development of advanced antimicrobial delivery platforms, which can enhance the efficacy, stability, and reusability of both existing and engineered antimicrobial agents. In this review, we provide an overview of the literature on the preclinical and clinical development of antimicrobial agents, as well as the integration of both existing and emerging agents into advanced delivery systems. These include inorganic nanoparticles (*e.g.*, metal- and silicon-based) as well as (supramolecular) organic nanoparticles such as polymeric micelles, dendrimers, vesicles, polyelectrolyte complexes, hydrogels, lipid micelles, liposomes, lipid nanoparticles, emulsions, and hybrid formulations, among others. Using a soft matter science perspective, we discuss these nanoparticle scaffolds as platforms for incorporating antimicrobial compounds via encapsulation, conjugation, adsorption, entrapment, self-assembly, co-assembly, or driven-assembly strategies. While many of these delivery systems show substantial promise, key challenges, including technical limitations, toxicity, and immunogenicity, must be addressed before clinical translation. By reviewing recent advances and innovations, we highlight how nanotechnology-enabled delivery systems can overcome challenges posed by AMR and potentially shape the future of antimicrobial therapies.

## Graphical abstract

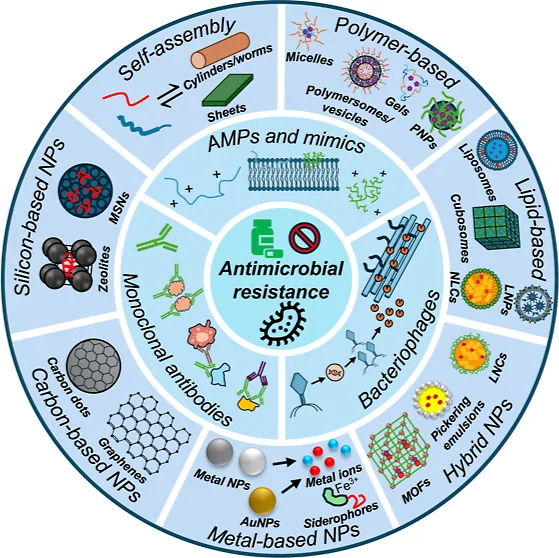

## 1 Introduction

Right after the commercialization of antibiotics in the mid-20th century, the war against bacterial infections was deemed over.^1,2^ The rapid evolutionary adaptability of bacteria, however, soon proved otherwise, undermining the long-term efficacy of these treatments.^1–3^ The fight against bacterial infections became a microbial arms race.^4^ When new antibiotics came to market, resistance quickly developed, creating an ongoing need to develop even more antibiotics.^1,5^ Antimicrobial resistance (AMR) arises from selective survival pressure, particularly when bacteria are exposed to (local) subinhibitory or subtherapeutic concentrations of antibiotics, *e.g.*, due to ineffective treatment management or misuse.^5–8^ As many of the prescribed antibiotics show low bioavailability and become less effective due to the presence of biofilms, *i.e.*, bacteria enclosed in a self-produced extracellular polymeric substance (EPS) matrix, higher or multiple doses are necessary to achieve adequate antibacterial activity, accelerating the development of resistance.^6,9^ Resistance can arise through *de novo* gene mutations but more commonly occurs via horizontal gene transfer (HGT).^5,10^ Moreover, plasmids are crucial in facilitating the exchange of genes between bacterial species, leading to the rapid spread of resistance genes across entire bacterial populations.^11,12^

In recent years, global awareness of the issue of AMR has grown.^8,13^ In 2019, the World Health Organization (WHO) estimated that 1.27 million deaths annually were attributable to drug-resistant bacterial infections, with morbidity and mortality rates expected to rise substantially in the coming years.^8^ If no measures are taken or no antimicrobial alternatives can be found, modeling projections suggest that by 2050, AMR could surpass cancer as a leading cause of death globally.^14^ As a result, several alternative strategies to conventional antibiotics are under active development and investigation.^15–20^ These include antimicrobial peptides (AMPs) and their synthetic mimics, *i.e.*, peptoids and antimicrobial polymers, as well as metal salts, siderophores, and biological approaches such as bacteriophage and monoclonal antibody (mAb) therapies. Additionally, nanoparticle-based antimicrobials (*e.g.*, metal-based and carbon-based nanoparticles) are primarily being explored for non-systemic applications, for example, as antimicrobial coatings. These alternative antimicrobial agents face challenges such as limited interest from pharmaceutical companies, high costs, low bioavailability, and cytotoxicity, hindering their ability to compete with established antibiotics.^15,17,21–25^

Although most of these alternative antimicrobial approaches are not yet ready for therapeutic use, they exhibit promising characteristics.^15,20^ These properties can be leveraged in the development of combination therapies by pairing them with commercial antibiotics.^24,26^ Additionally, they hold potential for integration into nanotechnology-based antimicrobial formulations, utilizing drug delivery platforms that employ encapsulation, conjugation, surface modification, or self-, co-, or driven-assembly strategies.^20,27–33^ These scaffolds can directly interact with bacteria/microbes or serve as a carrier of antimicrobial compounds.^27^ By designing nanomaterials and drug delivery systems capable of releasing antimicrobial compounds at a controlled time and place, their antimicrobial efficacy can be improved.^27,34^ This can lead to enhanced bioavailability and lower potential toxicity, factors that all improve the therapeutic index of antimicrobial agents while simultaneously reducing resistance development.^27,35,36^

Over the past decades, growing interest in combating AMR has driven significant efforts toward developing a diverse range of antimicrobial nanotechnology solutions. Antimicrobial compounds have already been integrated into a large variety of drug delivery platforms, such as inorganic (metal- and silicon-based) nanoparticles as well as supramolecular organic nanoparticles that can be based on polymers, lipids, surfactants, or hybrid systems. In this review, we discuss the progress in the field of antimicrobial nanoparticles, starting with an overview of existing commercialized antibiotics, resistance mechanisms, and emerging alternative antimicrobial agents currently under investigation. In this review, “antimicrobial” is used as an umbrella term for agents active against microorganisms, including bacteria and fungi, whereas “antibacterial” and “antibiotic” refer specifically to agents targeting bacteria, with “antibiotic” denoting clinically used antibacterial drugs. Our primary focus is on antibacterial therapy and resistance in drug-resistant bacterial pathogens, using the WHO bacterial priority pathogen list as the main clinical framework, while antifungal nanocarrier systems (*e.g.*, liposomal amphotericin B) are included only as illustrative case studies of nanomedicine translation to highlight design principles transferable to antibacterial nanoparticle-based formulations. We critically examine recent advancements in nanotechnology-based antimicrobial delivery strategies for both existing andrecently developed antimicrobial compounds, and conclude by highlighting the current state of this field and the limitations that must be overcome to translate these innovations into the antimicrobial compounds of the future.

## 2 Antibiotics and Resistance

### 2.1 Antibiotic Development and Commercialization

Over the last century, antibiotics have become one of the cornerstones of modern medicine.^1,37^ Following the discovery and development of penicillin and other early agents, the “golden era” of antibiotic discovery (1940–1960) yielded most of the major structural classes used today, including aminoglycosides, tetracyclines, polymyxins, cephalosporins, macrolides, glycopeptides, and rifamycins^1,38^ (Figure 1). However, soon after their introduction, it became apparent that bacteria rapidly acquire resistance, often within two to three years of clinical use.^1,39^

**Figure 1:**
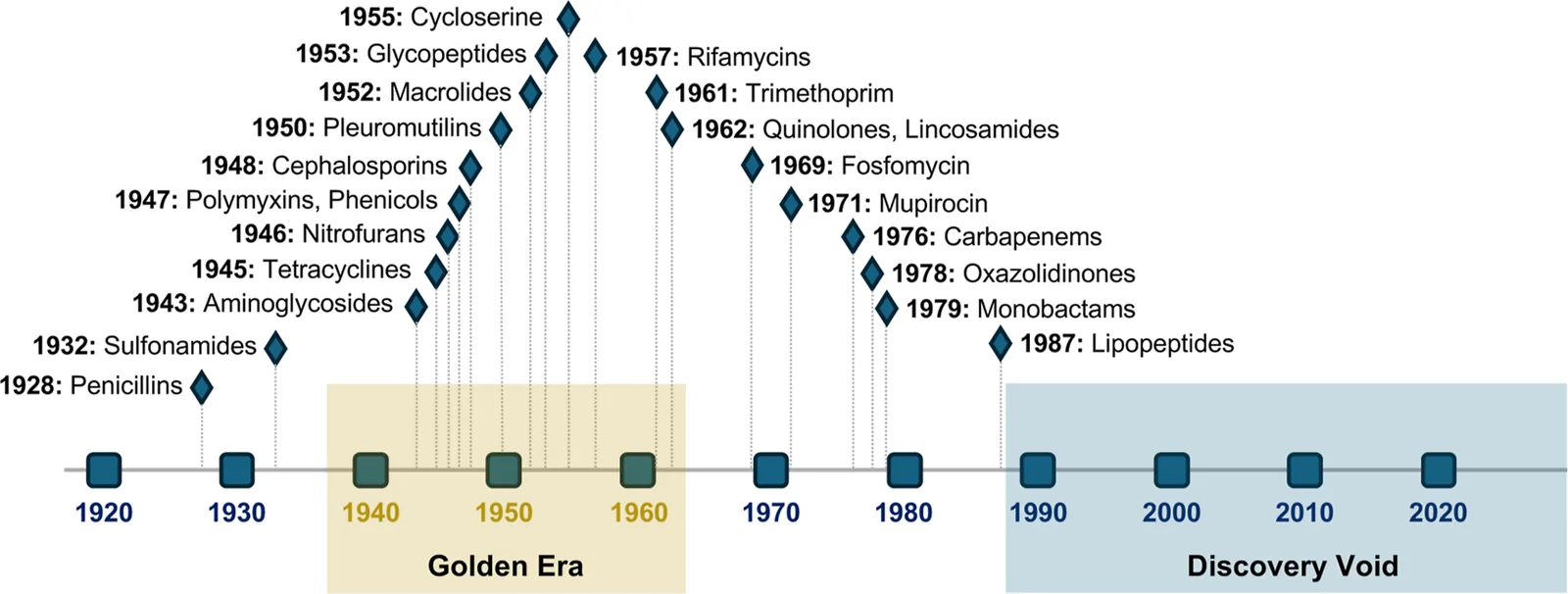
Timeline of the history of major antibiotic discoveries.^38,40^

In the following decades, the pace of antibiotic discovery declined as natural sources were exhausted, and many pharmaceutical companies withdrew, largely due to decreased profitability in the antibiotic development business.^1,2,41^ Even though, at a reduced pace, the discovery of the antibiotic groups of, *e.g.*, lincosamides (1962), carbapenems (1976), oxazolidinones (1978), and lipopeptides (1987), followed^38,42^ (Figure 1). In Table 1, the major antibiotic groups are listed and organized into their respective classes based on their mode of action.^15,43^ Notably, 17 of the 19 new antibiotics, with activity against WHO priority pathogens,^44^ that were approved for systemic use between 2017 and 2026, as of 10 August 2026, belong to these established structural groups (Table 2). This highlights the ongoing reliance on existing antibiotic classes for new drug development.^45,46^ The recent Food and Drug Administration (FDA; United States) approvals of gepotidacin and zoliflodacin in 2025 mark a rare breakthrough in antibiotic innovation with first-in-class antibacterial agents with novel mechanisms of action, after decades dominated by derivatives and combinations of established antibiotic classes.^47,48^ The remaining reliance on established classes highlights the constraints of conventional antibiotic discovery and underscores the need for alternative approaches to broaden the range of available antibacterial therapies. Nanotechnology-based formulations provide a complementary route to enhance the efficacy and therapeutic lifespan of these agents by optimizing delivery, targeting, and combination strategies.

**Table 1:** Classification of Major Commercialized Antibiotics Based on Their Mode of Action

mode of action	class	group	examples
cell wall synthesis inhibition	β-lactams	penicillins	amoxicillin, ampicillin, penicillin G, penicillin V
		cephems	cephalosporins (ceftriaxone, cephalexin, cefdinir), cephamycins
		carbapenems	meropenem, imipenem
		monobactams	aztreonam
	glycopeptides	glycopeptides	vancomycin, teicoplanin
		lipoglycopeptides	dalbavancin, oritavancin, telavancin
	miscellaneous		fosfomycin, cycloserine, bacitracin
protein synthesis inhibition	30S ribosome subunit inhibitors	aminoglycosides	gentamicin, amikacin, streptomycin
		tetracyclines	doxycycline, tetracycline, minocycline
	50S ribosome subunit inhibitors	macrolides	erythromycin, azithromycin
		lincosamides	clindamycin
		pleuromutilins	retapamulin, lefamulin
		oxazolidinones	linezolid
		amphenicols	chloramphenicol
	miscellaneous		mupirocin
nucleic acid synthesis inhibition	DNA gyrase inhibitors	quinolones	nalidixic acid
		fluoroquinolones	ciprofloxacin, levofloxacin, moxifloxacin
	RNA polymerase inhibitors	rifamycins	rifampin, rifabutin, rifapentine
	DNA damage inducers	nitroimidazoles	metronidazole, tinidazole
		nitrofurans	nitrofurantoin
metabolic pathway blockers	folic acid synthesis inhibitors	sulfonamides	sulfamethoxazole
	miscellaneous		trimethoprim, isoniazid, ethionamide, pyrazinamide
cell membrane integrity disruption	polypeptides	polymyxins	colistin, polymyxin B
		lipopeptides	daptomycin
		peptide antibiotics	gramicidin

**Table 2:** New Antibacterial Agents with Systemic Administration Routes Against Bacterial Species Included in the WHO Priority Pathogen-Framework^44^ Approved by Selected Major Regulatory Agencies after 2017 (Approval Status as of 10 August 2026)^a^

antibiotic	regulatory approval	year of approval	class (group)
delafloxacin	FDA/EMA	2017/2019	DNA gyrase inhibitors (fluoroquinolones)
meropenem/vaborbactam	FDA/EMA	2017/2018	β-lactams (carbapenems)
plazomicin	FDA	2018	30S ribosome subunit inhibitors (aminoglycosides)
omadacycline	FDA	2018	30S ribosome subunit inhibitors (tetracyclines; aminomethylcyclines)
eravacycline	FDA/EMA	2018/2018	30S ribosome subunit inhibitors (tetracyclines; fluorocyclines)
imipenem/cilastatin/relebactam	FDA/EMA/PMDA	2019/2020/2021	β-lactams (carbapenems)
lefamulin	FDA/EMA	2019/2020	50S ribosome subunit inhibitors (pleuromutilins)
pretomanid	FDA/EMA/CDSCO	2019/2020/2020	DNA damage inducers (nitroimidazoles)
lascufloxacin	PMDA	2019	DNA gyrase inhibitors (fluoroquinolones)
cefiderocol	FDA/EMA/PMDA/NMPA	2019/2020/2023/2026	β-lactams (cephems; siderophore)
levonadifloxacin	CDSCO	2019	DNA gyrase inhibitors (fluoroquinolones)
contezolid	NMPA	2021	50S ribosome subunit inhibitors (oxazolidinones)
sulbactam/durlobactam	FDA	2023	β-lactams (penicillins + β-lactamase inhibitor)
cefepime/enmetazobactam	FDA/EMA	2024/2024	β-lactams (cephems + β-lactamase inhibitor)
aztreonam/avibactam	EMA/FDA	2024/2025	β-lactams (monobactams + β-lactamase inhibitor)
sulopenem etzadroxil/probenecid	FDA	2024	β-lactams (penems + pharmacokinetic enhancer)
gepotidacin	FDA	2025	***novel*** **type II** **topoisomerase inhibitor (triazaacenaphthylene)**
zoliflodacin	FDA	2025	***novel*** **type II** **topoisomerase inhibitor (spiropyrimidinetrione)**
cefepime/zidebactam	FDA	2026	β-lactams (cephems + β-lactam enhancer)

a Regulatory approvals considered include the U.S. Food and Drug Administration (FDA; United States), European Medicines Agency (EMA; European Union), Pharmaceuticals and Medical Devices Agency (PMDA; Japan), Central Drugs Standard Control Organisation (CDSCO; India), and National Medical Products Administration (NMPA; China). Gepotidacin and zoliflodacin are highlighted in bold as first-in-class antibacterial agents with novel mechanisms of action.

### 2.2 Antibiotic Resistance

Extensive antibiotic use has increased the prevalence of antibiotic-resistant bacterial species throughout the years.^8^ In 2019, the six leading AMR pathogens responsible for 73% of the burden caused by AMR were: *Escherichia coli*, *Staphylococcus aureus*, *Klebsiella pneumoniae*, *Streptococcus pneumoniae*, *Acinetobacter baumannii*, and *Pseudomonas aeruginosa*. These were all classified by the WHO as priority pathogens, where carbapenem-resistant *A. baumannii* and carbapenem-resistant Enterobacterales and third-generation cephalosporin-resistant Enterobacterales received the “critical” status in 2024.^44^

AMR generally arises through intrinsic or acquired mechanisms.^5,10^ Intrinsic resistance is genetically encoded and relatively predictable, while acquired resistance occurs through mutations or HGT, making it less predictable.^5^ The latter can occur through three mechanisms: transduction (via bacteriophages), conjugation (plasmid transfer), and transformation (uptake of genetic material from lysed cells).^5,10^ Of these mechanisms, conjugation is considered an important driver in the spread of resistance, especially among Gram-negative bacteria.^49^ Under selective survival pressure, bacteria evolve resistance mechanisms that correspond to the mode of action of the antibiotics they encounter.^50–52^ One of the most widespread resistance mechanisms is enzymatic deactivation, where the bacteria produce enzymes such as β-lactamases, aminoglycoside-modifying enzymes, and chloramphenicol acetyltransferases that deactivate or break down a specific antibiotic structural moiety (Figure 2I).^51,53,54^ Other mechanisms include the increase of efflux pump activity (active antibiotic export), target molecule modification (mutations reducing antibiotic binding affinity), target molecule protection (proteins shielding antibiotic targets), reduced permeability (altered uptake through porin regulation), and physiological bypass mechanisms (alternative pathways for target replacement)^51,53–56^ (Figure 2).

**Figure 2:**
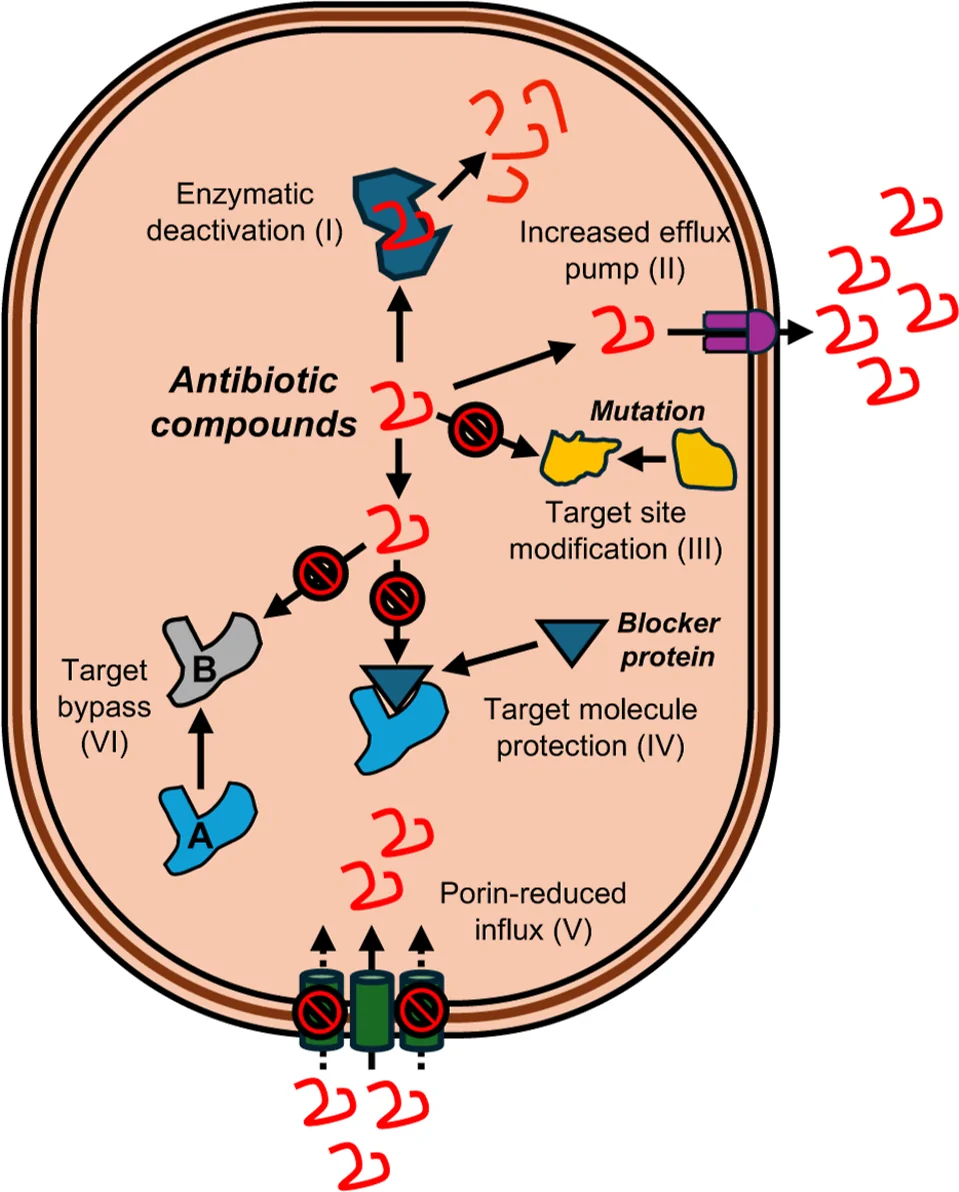
Molecular mechanisms of antimicrobial resistance in bacterial cells. (I) Enzymatic deactivation, where bacterial enzymes chemically modify or degrade antibiotics, rendering them inactive. (II) Increased efflux, in which efflux pumps export antibiotics from the cell, reducing intracellular antibiotic concentrations. (III) Target site modification, where mutations or chemical modifications decrease antibiotic binding affinity. (IV) Target protection, in which protective proteins shield the antibiotic target while preserving its biological function. (V) Reduced permeability, resulting from decreased uptake through altered or downregulated porins. (VI) Physiological bypass, where alternative enzymes or metabolic pathways replace or bypass the inhibited target.

Biofilms play a crucial role in the development of resistance, with around 80% of infections in hospitalized patients occurring in biofilm-associated forms.^57,58^ Biofilms are aggregates of microbial cells encased in a self-produced (hydrogel-like) matrix of extracellular polymeric substances (EPS) that often attach to surfaces or host cells, or are found within the mucosal layer.^57,59,60^ Cells embedded in biofilms exhibit significantly lower sensitivity to antibiotics compared to their planktonic counterparts (free-floating bacterial cells), exhibiting up to 1000 times greater tolerance to antibiotics.^59,60^ These effects are caused by a combination of their slower growth rate, limited penetration of antibiotics, and reduced bacterial metabolic activity within the biofilm.^59,61^ Biofilms can also act as a source of infection when they can grow on medical devices.^58,62^ Therefore, strategies to reduce or control biofilm formation are essential in the development of antibiotic approaches.^62^

## 3 Emerging Antibacterial Compounds with Reduced AMR Susceptibility

Documenting the developments of emerging antibacterial agents, the WHO reported that, as of February 2025, 90 products were in clinical stages focusing on targeting bacterial priority pathogens.^46,63^ These include 50 so-called traditional antibiotics and 40 non-traditional antibacterials. Almost half of the traditional antibiotics currently in the antibiotic discovery pipelines belong to the category of β-lactams, β-lactamase inhibitors, or combination therapies thereof, which aim to enhance efficacy through synergistic effects of multiple components.^63^ Combination treatments are increasingly favored (Table 2), as they offer an often more cost-effective alternative in improving antibiotic effectiveness, at reduced toxicity and lower risk of resistance development, while broadening antibacterial coverage compared to single-drug therapies.^64^ Other traditional antibiotics currently under investigation mostly fall within the groups of widely commercialized antibiotics, such as tetracyclines, macrolides, and aminoglycosides, which, due to their lower degree of novelty and higher susceptibility to resistance development, will not be discussed further in this review (Table 1).

Instead, in this section, we provide an overview of the most emerging categories of antibacterial agents currently under development. We focus on new cell membrane-disrupting antibacterials, bacteriophage-based and antibody-based therapies, and their potential shown from preclinical and clinical investigations. These agents, due to their different modes of action, are often less susceptible to the molecular mechanisms of antibiotic resistance (Figure 2). Although very few of these have reached stage III clinical trials, many hold promise for addressing multidrug-resistant infections, and further advancements in clinical development are expected in the coming years. Additionally, their distinctive mechanisms of action and often challenging pharmacokinetic profiles create clear opportunities for nanoparticle-enabled delivery and combination therapies, which we discuss in later sections.

### 3.1 Antimicrobial Peptides and Their Mimics

Recent WHO reports highlight significant progress in the development of (synthetic) cell membrane-disrupting antibiotics inspired by antimicrobial peptides (AMPs), including engineered polymyxin analogs and macrocyclic peptides.^63^ These AMPs, sometimes referred to as host-defense peptides (HDP), have increasingly gained attention over the last couple of decades.^65,66^ AMPs typically consist of 10–50 amino acids and play a crucial role in the innate defense mechanisms across different forms of life.^66^ Unlike conventional antibiotics, AMPs exhibit broad-spectrum activity by targeting bacterial membranes across multiple groups while also possessing the ability to eliminate viruses and fungi.^65^ Most AMPs are cationic and amphiphilic, contributing to their structural diversity and enabling them to function in both unfolded and folded/assembled states^65–67^ (Figure 3). Their activity is often influenced by interactions with biological membranes, which can induce conformational changes.

**Figure 3:**
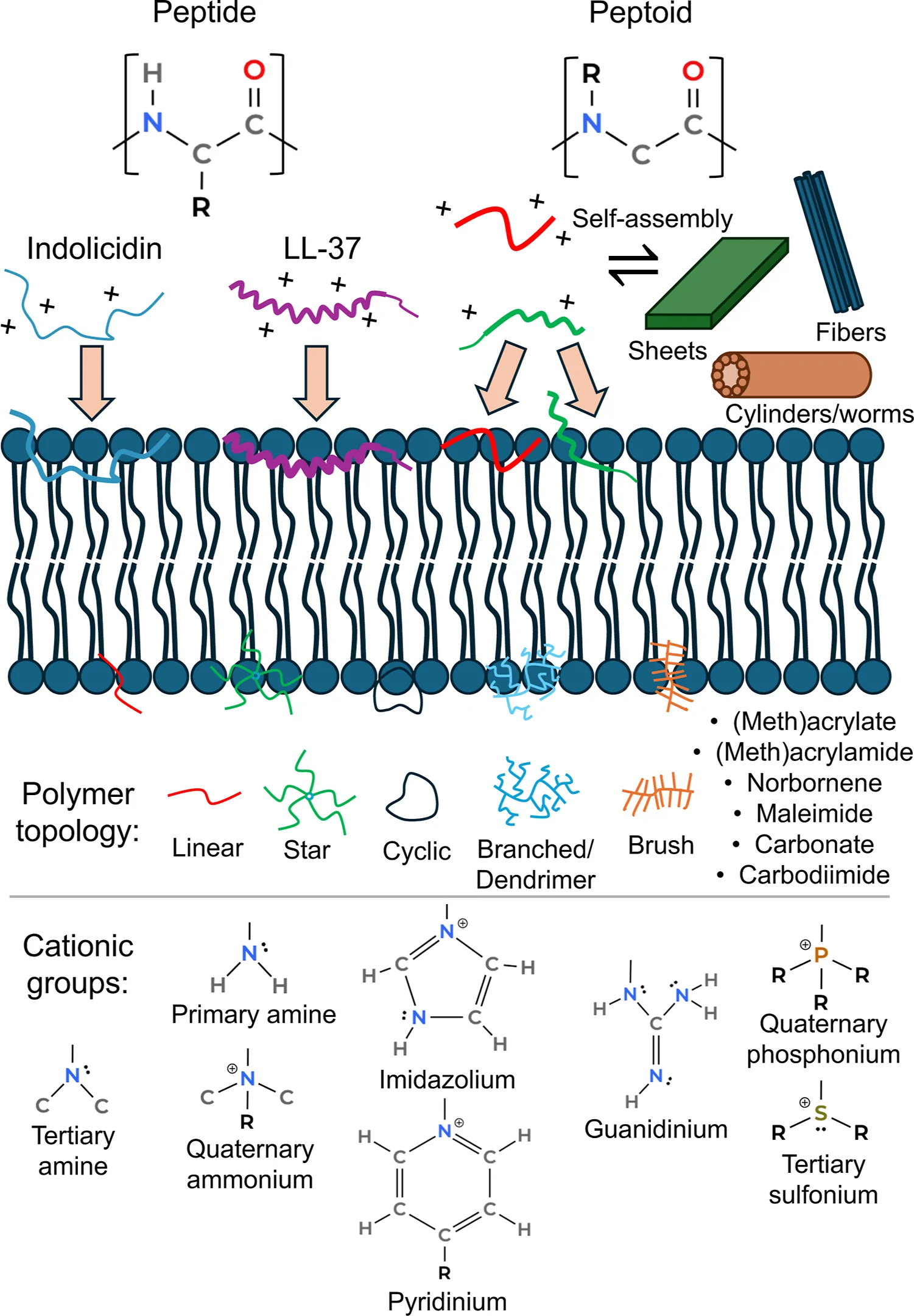
Antimicrobial peptides and their mimics/derivatives. AMPs, such as indolicidin and LL-37, are known to insert into the membrane as depicted.^68^ Antimicrobial peptoids are synthetic peptidomimetics that are often found to self-assemble into several structures. Peptoids interact similarly with the bacterial membrane in monomeric form. The widest category of AMP mimics is the antimicrobial polymers that can be synthesized in many forms with different cationic groups. They are thought to also interact with the bacterial membrane through insertion. The molecular structures of the cationic groups and the peptides and peptoids are illustrated using the tool from MolView. The peptide and peptoid structures are illustrated based on PDB structures.

In 1939, Gramicidin was the first AMP to be discovered, isolated from the soil bacterium *Brevibacillus brevis*.^69^ In the following decades, other bacterial-derived compounds, such as polymyxins (including colistin, also known as polymyxin E, and polymyxin B) (1940s and 1950s) and lipopeptides like daptomycin (1980s), were also recognized as part of the group of AMPs^38,70,71^ (Figure 1). Following the depletion of the bacterial-derived antibiotic sources during the 1980s and 1990s, there was a growing interest in immuno-active AMPs of animal origin.^71^ This led to the discovery of several natural AMPs, such as cecropin (moth-origin), LL-37 (human), indolicidin (bovine), aurein (frog), and magainin (frog), among many others^71,72^ (Figure 3). The further identification and characterization of AMPs have led to the discovery of over 3300 different natural AMPs to date, with many originating from diverse organisms across all domains of life.^73,74^

The clinical development and use of AMPs present several challenges, including high development and production costs, limited efficacy in therapeutic settings, cytotoxicity, and, even though with lower prevalence, the potential for resistance to emerge.^23,69,75,76^ Additionally, their bactericidal mode of action is not fully understood and varies largely for each peptide structure.^68,75^ Generally, AMPs interact with bacterial cell membranes, leading to destabilization and triggering bacterial cell death.^68^ The most commonly discussed “classical” theories regarding these interactions involve transmembrane pore formation, where AMPs self-assemble into pore-like structures, and membrane solubilization, in which AMPs act as surfactants.^75,77^ However, the literature on these pore structures remains scarce and debated, while surfactant behavior is often only observed at supraphysiologically high concentrations.^78^ More recent studies have provided evidence for alternative mechanisms, including AMPs promoting lipid flip-flop (*trans*-bilayer movements)^68,79–81^ and inducing membrane thinning or thickening.^75,82^ These processes can lead to in-plane phase separation, facilitating transient pore formation, water channeling, and significantly reducing membrane stability.^68,75^ Other AMPs, such as polymyxins (colistin and polymyxin B) and daptomycin, exhibit distinct mechanisms of action while still targeting the bacterial membrane.^68^ Polymyxins are known to interact with the hydrophobic region of Lipid A in the lipopolysaccharide (LPS) layer,^83,84^ whereas daptomycin is known to cause a calcium-mediated interaction with the membrane, resulting in ion fluxes across the membrane.^85,86^

AMPs have been shown to reduce biofilm formation for multiple bacterial strains.^87^ Specifically, LL-37 and indolicidin have been reported to downregulate the transcription of cellular signals that regulate quorum-sensing systems in *P. aeruginosa*, thereby inhibiting biofilm development.^87,88^ Despite showing promising characteristics, the negatively charged polymers in the biofilm matrix could interfere with the positively charged AMPs, reducing their diffusion and antimicrobial activity.^89^

Although AMPs are generally less likely to induce resistance in bacterial populations, resistance has still been observed for several AMPs.^90,91^ Gram-negative bacteria have been found to utilize multiple strategies to defend themselves against polymyxins, including various modifications of their LPS layer, excretion of negatively charged polymers to bind the polymyxins (capsular polysaccharides, seen in *K. pneumoniae*), and efflux pump up-regulation^65,92^ (Figure 4B,D,E). For daptomycin, which targets Gram-positive infections, resistance has been reported based on cell envelope modifications like d-alanine modification and the incorporation of basic amino acids in the membrane, illustrated in Figure 4A,C.^65,93^ Similar resistance mechanisms have also been observed for non-therapeutic AMPs, such as LL-37, PR-39, cecropin, and magainin,^94–98^ including degradation by bacterial proteases, like elastase from *P. aeruginosa* ^99^ (Figure 4F).

**Figure 4:**
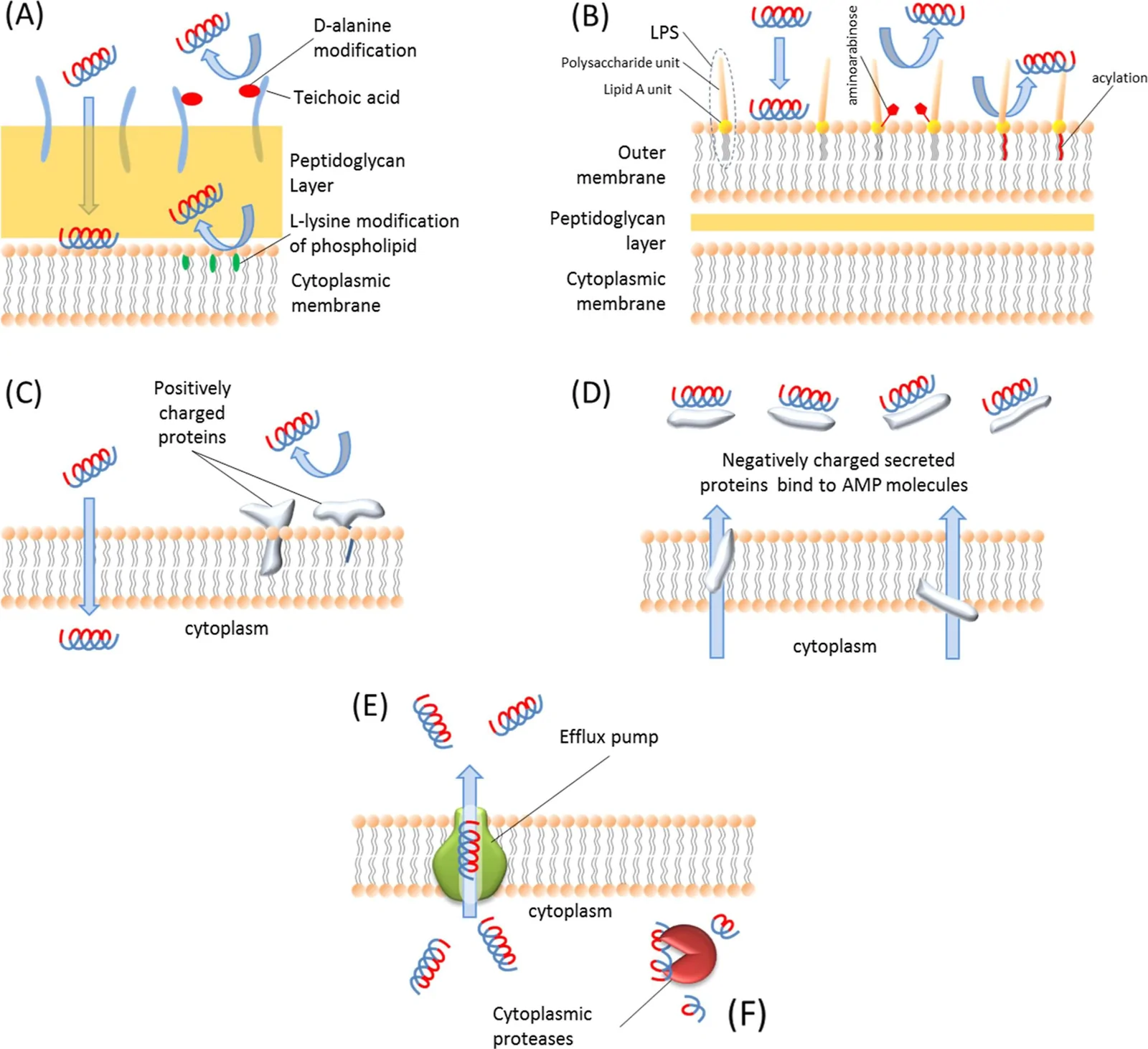
Molecular mechanisms of bacterial resistance towards antimicrobial peptides (AMPs). Gram-positive bacteria resist AMPs by modifying teichoic acids in their cell walls and altering phospholipids through l-lysine modification (A). Gram-negative bacteria evade AMPs by modifying lipopolysaccharide (LPS) molecules with aminoarabinose or acylating the Lipid A unit (B). Some bacteria incorporate positively charged proteins into their membranes, creating charge repulsion that prevents AMPs from binding effectively (C). Certain bacteria secrete negatively charged proteins into the extracellular environment, where they bind to and neutralize AMPs (D). Intracellular AMPs are actively removed by efflux pumps (E). AMPs that enter the cell are degraded by bacterial proteases (F). The figure is reproduced from Bahar & Ren (2013)^65^ under the terms of the Creative Commons Attribution License (CC BY 3.0).

Although bacteria have evolved multiple strategies to counteract AMPs, the universal presence of lipid bilayer membranes makes it difficult for them to achieve complete resistance.^65,99^ Importantly, resistance to AMPs has so far been less pronounced than to conventional antibiotics and is generally confined to a limited subset of AMPs. Differences in pharmacodynamics further support this, as AMPs often display steeper dose–response curves, suggesting a reduced likelihood of resistance evolution.^76,90^ Nevertheless, their limited proteolytic stability remains a major obstacle for clinical application.^100,101^ To date, only few natural AMPs have advanced into clinical development, since their therapeutic potential is restricted by challenges such as cytotoxicity and low bioavailability.^22^ To mitigate these side effects, due to their relatively simple peptide structure, synthetic versions can be developed, showing higher stability and lower toxicity.^76^ This can, for example, involve a change of sequence,^67,102^ development of ultra-short AMPs,^103–105^ the addition of unnatural amino acids (d-amino acids),^100^ but also the synthesis of synthetic mimics, such as antimicrobial peptoids and polymers.^31,102,106–108^

A particularly recent breakthrough in AMP development was the approval of peceleganan (PL-5), a synthetic antimicrobial peptide for topical use, by the Chinese National Medical Products Administration (NMPA) in June 2026. Approved for the treatment of burn-wound infections, peceleganan represents a notable advancement in the field of AMPs, particularly for localized infections where direct topical administration can provide high local antimicrobial activity while limiting systemic exposure.^109,110^ Several other synthetic AMP derivatives are currently in clinical trials as antimicrobial agents that target priority pathogens.^46,63^ These include murepavadin, a protegrin-1 mimic,^111^ PLG0206, an investigational anti-biofilm peptide being studied for the treatment of periprosthetic joint infections,^112^ OMN6, a cecropin derivative, which recently progressed to phase IIa,^46,113^ and polymyxin analogs like upleganan, MRX-8, and QPX9003.^46,63,114,115^ For the most current information on AMPs in clinical trials, including their progress and development stage, it is advised to consult updated clinical trial databases.^63,116,117^

Despite extensive investigation and potent antimicrobial activity demonstrated by many AMPs, the clinical translation into systemically administered antibacterial therapeutics of even well-characterized peptides remains limited by challenges including susceptibility to degradation, poor pharmacokinetic properties, and potential toxicity. To address these challenges, nanoparticle-based strategies have increasingly been explored to improve AMP stability, enhance delivery to infection sites, and reduce off-target effects. These approaches include both AMP-loaded and AMP-functionalized nanoparticles, such as metal nanoparticles, inorganic NPs, and polymer-, lipid-, and hybrid-based nanocarriers. The integration of AMPs with these nanoparticle platforms represents a promising approach to enhance their therapeutic potential. Relevant examples are therefore cross-referenced and discussed in more detail in Sections 4 and 5.

#### 3.1.1 Antimicrobial Peptoids

Peptoids, *i.e.*, poly-*N*-substituted glycines, resist proteolysis and enzymatic degradation due to their side chain positioning^101,118^ (Figure 3). Moreover, the side chain groups (R) are attached to the amide nitrogen atom rather than the α-carbon. Antimicrobial peptoids, designed to mimic natural AMPs, not only exhibit enhanced stability but also often display promising antimicrobial activity, including effectiveness against multidrug-resistant (MDR) bacteria.^119–124^ Furthermore, they are more cost-effective as they are easier to synthesize than conventional α-peptides.^118^ In some cases, antimicrobial peptoids also exhibit greater efficacy with lower toxicity compared to natural AMPs.^68,125,126^

Antimicrobial peptoids are thought to function similarly to AMPs, where their cationic regions (electrostatically) interact, depolarize, and disrupt anionic bacterial membranes.^127^ However, the bactericidal activity of peptoids may also arise from the nonspecific aggregation of intracellular components, contributing significantly to bacterial cell disruption.^120^ In addition to the chemical structure, the self-assembly of peptoids also needs to be considered when assessing their biological activity^31,128^ (Figure 3). Antimicrobial peptoids have been shown to self-assemble into diverse supramolecular morphologies such as helical bundles, core–shell ellipsoidal micelles, worm-like micelles, nanofibers, sheets, and globular clusters.^31,127,128^ Peptoids with self-assembly properties can show improved stability and offer benefits such as sustained drug release protection towards enzymatic degrand the potential to serve as carriers for other therapeutic agents^126^ (see Section 5). However, it is most likely that, regardless of their supramolecular assembly, peptoids interact with the membrane as monomer units, with their antimicrobial activity potentially depending on the release of monomers upon contact with lipid bilayers.^129^ Although antimicrobial peptoids possess promising properties and are readily synthesized, research remains largely at the preclinical stage, and further studies are needed to fully explore their potential as antimicrobial agents. Their propensity for controlled self-assembly and the ease of chemical modification make peptoids particularly attractive building blocks for nanoscale formulations, including peptoid-based nanoparticles and hybrid nanocarriers, which can enhance stability and allow co-delivery with conventional antibiotics.

#### 3.1.2 Antimicrobial Polymers

In contrast to antimicrobial peptoids, the field of antimicrobial polymers is older and more established.^130^ Polymers designed to mimic natural AMPs often include backbones of methacrylates, norbornenes, and methacrylamides, with cationic groups based on primary amines (−NH_2_), *e.g.*, lysine analogs such as aminopropyl methacrylamide (APMA) or guanidino groups (HNC­(NH_2_)_2_, *e.g.*, arginine analogs like guanadinopropyl methacrylamide (GPMA)^131^ (Figure 3). The cationic charge can also be incorporated into polymer structures through imidazolium salts, pyridinium salts, quaternary phosphonium salts, quaternary ammonium salts, or tertiary sulfonium salts. By modifying and controlling polymer topology, antimicrobial properties can be fine-tuned through structures such as linear homopolymers and copolymers, cyclic polymers, and advanced architectures like dendritic, star-shaped, or hyperbranched polymers.^132^ These structural variations influence charge distribution, hydrophobicity, and molecular interactions, enhancing bacterial membrane disruption, stability, and bioavailability.^108,132^ Owing to their structural versatility, antimicrobial polymers have both systemic and non-systemic applications, such as surface coatings, and can, due to their adaptable material properties, be customized for a wide range of uses. Similar to AMPs, these polymers often function by disrupting bacterial membranes through electrostatic interactions.^131,132^ In addition to disrupting bacterial membranes, antimicrobial polymers can inhibit enzymes and prevent biofilm formation, likely through interference with bacterial communication systems, like quorum sensing.^108,133–135^ Although antimicrobial polymers are currently limited to preclinical studies, their broad chemical diversity allows for the development of innovative antimicrobial strategies, making them a promising platform for future therapeutic applications as antimicrobial agents.^135^ Beyond their direct antimicrobial activity, the tunable architecture and surface chemistry of these polymers enable their use as nanoparticle matrices, coatings, and functional shells. This dual role, as active agents and as structural components of nanocarriers, creates important opportunities for designing polymer-based nanoparticles that synergize with traditional antibiotics, as discussed in Section 5.2.

### 3.2 Bacteriophage Therapy

Bacteriophages, also known as phages, are viruses that specifically infect certain bacterial species or strains^136,137^ (Figure 5). These phages are small (generally 50–200 nm) and require a specific bacterial host for reproduction.^136^ Unlike most antibiotics, they selectively target harmful bacteria without affecting human cells or other beneficial bacteria, making them a promising approach for treating bacterial infections.^138^ Phage therapy is far from new, as its development started in the 1920s and was quickly further developed during the Second World War.^139,140^ In the Soviet Union, which became isolated from Western antibiotic developments during and after the war, phage therapy continued to develop independently.^139^ Following the Cold War, interest in phage therapy resurged in the West as part of efforts to discover novel antimicrobial compounds.^140^ Nowadays, phages are only routinely used in Georgia and several former Soviet countries, but their clinical application in Western countries has not been widely authorized.^141^ However, as the reports of the clinical results of phage therapy are promising, particularly on multidrug-resistant infections, their clinical use is currently widely researched.^63,136,137^ As of February 2025, the WHO reported that phage and phage-derived therapeutics represented roughly half of the non-traditional antibiotic treatments in the clinical pipeline in the fight against priority pathogens.^46^

**Figure 5:**
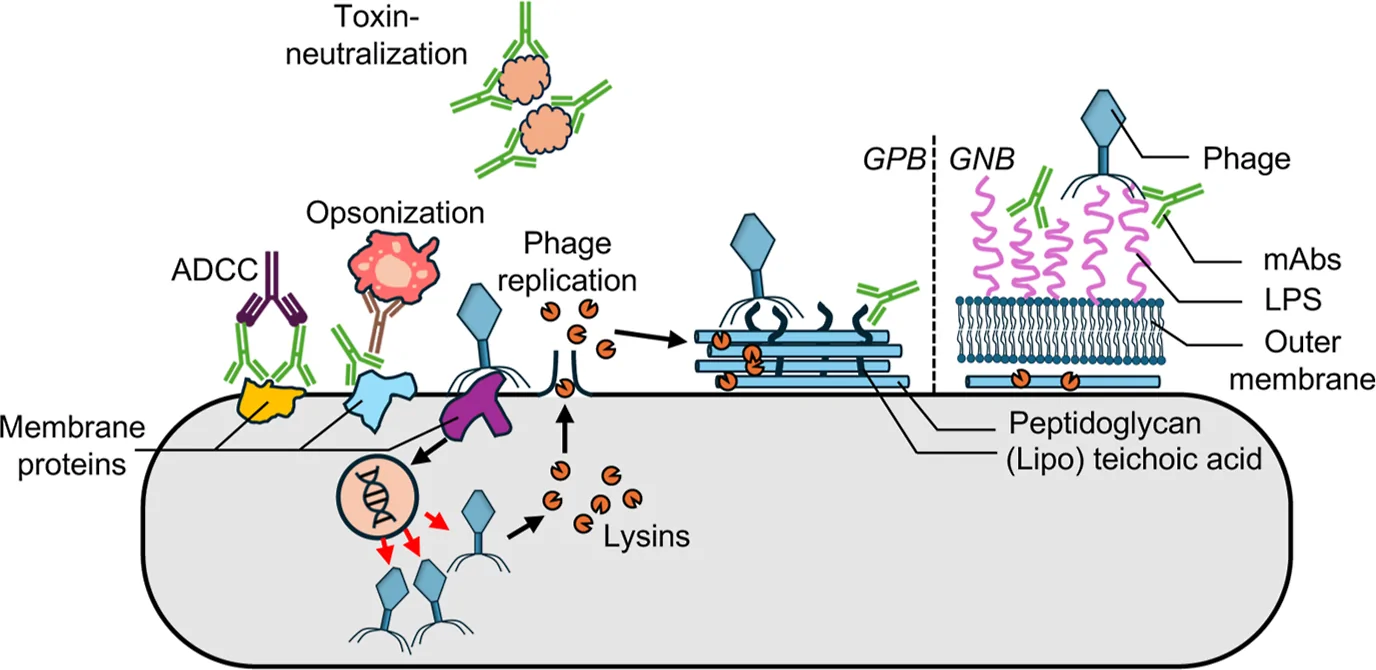
Antibacterial activities and pathways of bacteriophages (phages) and monoclonal antibodies. Phages recognize and bind to specific components on the bacterial cell surface, such as lipopolysaccharides, (lipo)­teichoic acids, and membrane proteins. They inject their genetic material into the host, which can either integrate into the bacterial genome (lysogenic cycle) or initiate production of phage enzymes, including lysins (lytic cycle). Lysins degrade the peptidoglycan layer, causing bacterial cell lysis and release of new phages. mAbs also bind to LPS, (lipo)­teichoic acids, and membrane proteins, but most often have antibacterial activity through the neutralization of toxins. When mAbs bind to membrane proteins, they can trigger opsonization (killing by macrophages) and antibody-dependent cellular cytotoxicity (ADCC) from an immune-mediated response. On the left, the most important antibacterial mechanisms are illustrated, while on the right, the differences in binding sites between Gram-negative bacteria and Gram-positive bacteria are depicted.

Phage therapy can generally be used in three ways: (1) using phage-derived lysins, which are enzymes that break down bacterial cell walls, (2) selecting specific phages to treat drug-resistant infections in individual patients, and (3) using predefined phage cocktails to broaden the host range for more effective treatment.^136,137,142^ Phages use their receptor-binding proteins (RBPs) to attach to bacterial surface structures such as lipopolysaccharides (LPS), teichoic acids (specific to Gram-positive bacteria), flagella, or membrane proteins to “infect” (lysogenic cycle) or infection followed by cell death (lytic cycle)^143,144^ (Figure 5). Upon attachment, phages inject their genetic material into the bacterial cell. While some phages naturally carry genes encoding toxins or enzymes, therapeutic phages are selected to avoid toxin transfer, and the lytic cycle is preferred to directly induce bacterial cell death.^137^ Phages are also effective against biofilms: they can penetrate the extracellular matrix, infect bacteria within, and disrupt the biofilm structure.^137,145^ Beyond conventional therapy, phages have been explored as vehicles for delivering enzymes or antibiotics.^137,146^

Their abilities as encapsulation and delivery vehicles can be used for therapeutic gene delivery, which can include gene editing via CRISPR-Cas systems.^137^ This gene editing is also used to optimize the efficacy of the phages themselves.^136,137^ Very recently, so-called “engineered phages” have emerged as an area of interest, which refers to phages that have been genetically modified through the removal or addition of genes.^136^ In 2019, the first successful case treatment with engineered phages was reported by Dedrick et al. (2019), where they cured a *Mycobacterium abscessus* infection through repressor gene deletion in bacteriophages, resulting in the conversion of lysogenic to lytic phages, allowing for more effective bacterial eradication.^136,147^

Even though their discovery already occurred more than a century ago, phage therapy still has some challenges to overcome before it can be widely used in clinical settings.^137,148^ Key issues include the specificity of a bacterial infection, host immune responses, and intracellular delivery.^137,149^ Additionally, the risk of bacterial resistance through induced horizontal gene transfer should be reduced, and the possibility of phage genes integrating into bacterial DNA, potentially increasing virulence, needs to be monitored.^137^ To address some of these limitations, recent research has explored the incorporation of bacteriophages into drug delivery platforms, both polymer- and lipid-based.^150^ These approaches aim to enhance the stability, targeting, and intracellular access of phages, while also offering opportunities to combine them with antibiotics or other therapeutic agents. Although bacteriophages are not yet widely approved for clinical use, they are likely to become a key tool in the future arsenal of antibiotic therapies.

### 3.3 Antibacterial Monoclonal Antibodies

The last major category of emerging non-traditional antibacterial agents is monoclonal antibodies (mAbs)^24,63,151^ (Figure 5). mAbs are antibodies produced by cloning a white blood cell from a single cell line. Antibody therapies that target bacterial pathogens, specifically from the IgG class of antibodies, have recently obtained more attention following their success in treating cancer.^63,151,152^ As of 2026, three antibacterial mAbs have been approved by the FDA: raxibacumab, obiltoxaximab, and bezlotoxumab.^153,154^ All FDA-approved mAbs have a mode of action based on toxin neutralization and are therefore dependent on other antibiotics for infection eradication.^151,154^ Various mAbs targeting priority pathogens are currently in clinical trials, with diverse modes of action mechanisms targeting multiple different target bacteria.^46,63^

In addition to neutralizing antigens and toxins, several mAbs under preclinical and clinical development can directly attack bacteria through antibody-dependent cellular cytotoxicity (ADCC) and opsonization, enhancing cytotoxicity and promoting phagocytosis^151,155,156^ (Figure 5). Antibody binding sites include lipopolysaccharides (LPS), teichoic acids, capsular polysaccharides, and bacterial membrane proteins.^154,157^ mAbs can also target quorum-sensing molecules and EPS elements, allowing them to disrupt and combat bacterial biofilms.^151,154,158^

The development of effective mAb treatments has been highly challenging.^151^ Many clinical trials have failed due to the discrepancies in the translation from preclinical animal models to human clinical trials.^159,160^ Moreover, these mAbs have been shown to result in immunogenic side effects if they are not humanized, resulting in high development costs.^157,161^ Additionally, once approved as a drug, their clinical use has been proven difficult. Due to their high specificity, mAbs suffer not only from the requirement of correct pathogen identification but also from the heterogeneity of that specific pathogen within its population.^162^ This specificity is a significant advantage, as it allows mAbs to effectively combat bacterial infections while possessing a low probability of resistance development and cytotoxicity.^155,161^ However, resistance can still emerge through mechanisms such as protease activity, target modification, or target blockage.^163,164^ Even though mAbs have some drawbacks, their success in battling cancer has proven their excellent targeting properties.^152,165^ Learning from this, their applicability has significant potential, especially combined with chemical engineering and drug delivery applications.^152,166^ For example, antibody-conjugated nanomaterials and mAb-based drug delivery approaches (discussed in Section 5) offer significant potential for the development of antibiotics in the future.^166–168^

## 4 Metal-, Carbon-, and Silicon-Based Antimicrobials

The growing challenge of AMR has prompted the exploration of a broader and more diverse set of antimicrobial strategies.^17,18,169–171^ Among these, antimicrobial nanoparticles and coatings employing metal-, carbon-, and silicon-based agents have received significant attention over the last few decades. These materials can offer distinct modes of action compared to conventional antibiotics, such as the generation of reactive oxygen species, membrane disruption, metal ion release, and/or physical puncture, modes of action that potentially have a reduced likelihood of resistance development^16,169,170,172,173^ (Figure 6). Additionally, they can act as efficient carriers, improving the delivery and performance of existing antibiotics. Metal-based nanoparticles have already demonstrated broad-spectrum antimicrobial activity and have been incorporated into textiles and as coatings on medical devices and other surfaces.^17^ In contrast, carbon-based nanoparticles show promising characteristics for material coatings, but for now, their application largely remains at the experimental stage.^18^ In addition to non-systemic applications, some metal- and silicon-based antimicrobials, such as siderophore-based antibiotics, metal complexes, metal-antibiotic conjugates, mesoporous silica nanoparticles (MSNs), and zeolites, have been the subject of preclinical and clinical investigation for systemic applications.^171,174–176^ In this section, we provide an overview of the development of metal-, carbon-, and silicon-based antimicrobials for both non-systemic and systemic administration routes.

**Figure 6:**
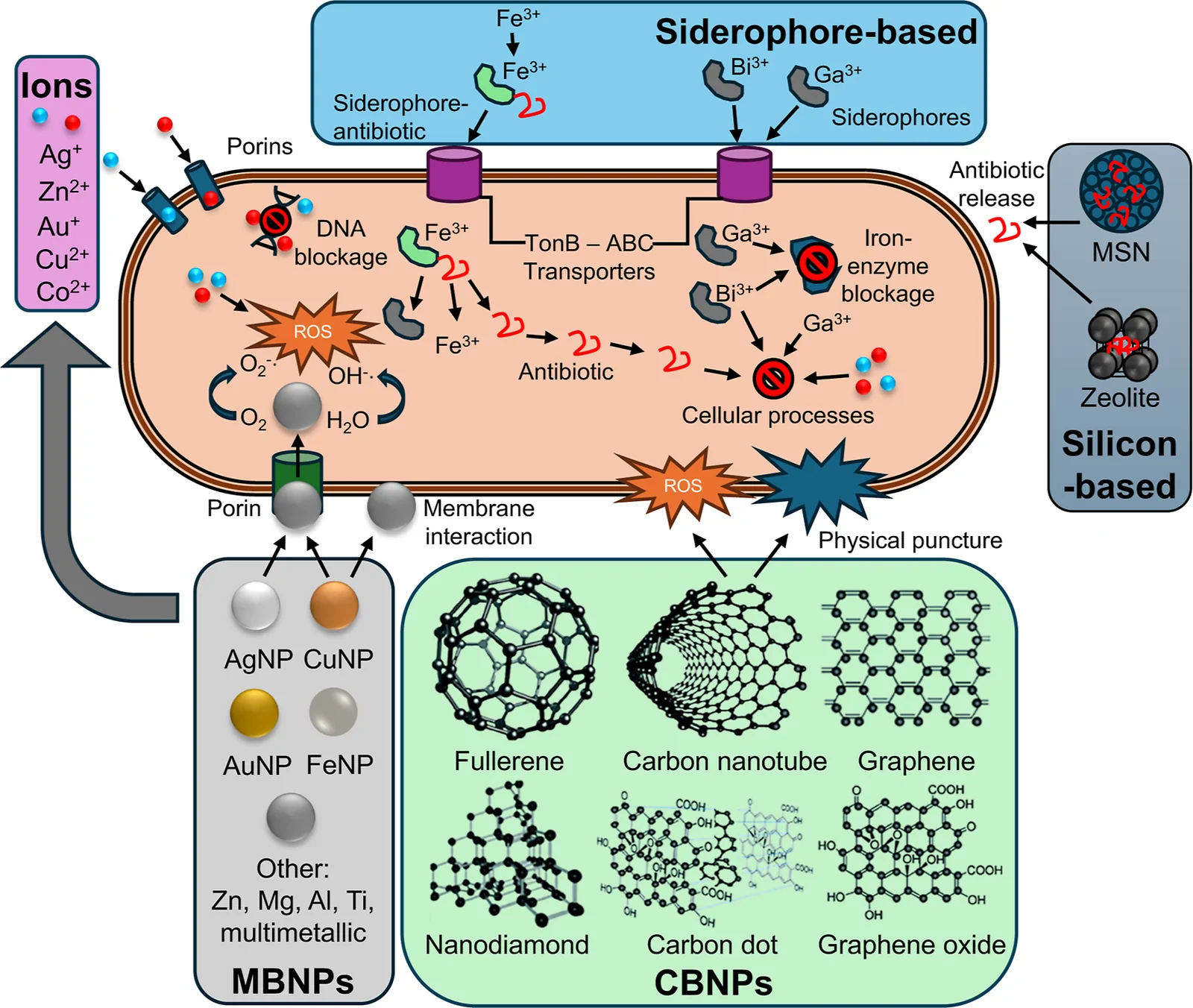
Antimicrobial agents and nanoparticles based on metals, siderophores, carbon, and silicon materials exert their effects on bacterial cells through distinct mechanisms. Carbon nanoparticles primarily disrupt bacterial membranes via mechanical disruption of the bacterial membrane (physical puncture) and induce oxidative stress by generating reactive oxygen species. Metal-based nanoparticles and metal ions contribute to ROS production and physically interact with the outer membrane. However, they primarily enter bacterial cells through porins, where they disrupt cellular functions and inhibit DNA replication. Siderophores facilitate the uptake of iron, as well as bismuth and gallium ions, leading to the disruption of multiple cellular processes, including enzyme inhibition and iron homeostasis. Furthermore, siderophore-antibiotic conjugates can exploit the TonB and ABC transporter pathways to enhance antibiotic delivery into bacterial cells, thereby targeting essential cellular functions. Silicon-based nanoparticles, including mesoporous silica nanoparticles and zeolites, can release antimicrobial agents that are encapsulated within or conjugated onto their structures, allowing the drugs to exert their therapeutic effect. The carbon-based nanoparticle illustrations are adapted from Yan et al. (2016)^181^ under the terms of the Creative Commons Attribution License (CC BY 3.0).

### 4.1 Metal-Based Nanoparticles and Metal Salts

Metal-based nanoparticles (MBNPs) offer a promising alternative to combat the growing resistance to conventional antibiotics and are among the most widely studied inorganic nanoparticles.^17,177^ Long before the boom of pharmaceutical antibiotics, metals were already used to combat bacterial infections.^178^ Nowadays, metals are used as antimicrobial agents in the form of bulk materials, metal salts, and nanoparticles.^17^ Metal salts are active through the release of ions like silver (Ag^+^), zinc (Zn^2+^), and copper (Cu^2+^) that enter (bacterial) cells through porins and exert their antimicrobial effect through facilitating DNA blockage, blocking other cellular processes, and generation of reactive oxygen species (ROS) (Figure 6). These salts are commonly used in coatings for medical devices and wound care products.^179,180^

In nanoparticle form, these metals have a greater antimicrobial effect due to substantial increases in surface area.^177^ Therefore, most of the research has been focusing on nanoparticles of various metals and their oxides with potent antimicrobial activity, including silver (Ag), gold (Au), copper (Cu), copper oxide (CuO), zinc oxide (ZnO), aluminum oxide (Al_2_O_3_), magnesium oxide (MgO), titanium dioxide (TiO_2_), and iron oxide (Fe_3_O_4_)^16,182–185^ (Figure 6).

They can also be developed as combination therapies, where traditional antibiotics and antimicrobial peptides^186–188^ are conjugated (functionalized) to the surface of these particles to improve their properties through synergistic effects.^169,189,190^ Alternatively, combinations of metals, called bimetallic or multimetallic NPs, have emerged as another strategy to enhance antimicrobial performance.^16,191^ MBNPs exert antimicrobial effects through multiple mechanisms, including the release of metal ions that induce oxidative stress, inhibiting enzymes, and interfering with DNA replication. In addition, the nanoparticles can interact directly with the bacterial membrane or enter the cell, further contributing to oxidative stress and cellular disruption^16,172,192^ (Figure 6).

By employing different synthesis techniques, MBNPs can be produced in various morphologies and sizes.^16,193^ The size of the MBNPs has been reported to have a significant influence on the antimicrobial mechanism and action, with smaller-sized particles having a lower minimum inhibitory concentration (MIC).^17^ Bimetallic nanoparticles containing combinations such as CuAg and AuAg have recently regained interest due to their potential to overcome the limitations of single-metal antimicrobial agents, including insufficient antimicrobial potency, limited control over ion release, and potential toxicity.^191,193,194^ The simultaneous release of different metal ions can produce synergistic antimicrobial effects by combining complementary mechanisms of action and may reduce the risk of bacterial adaptation compared with single-metal nanoparticles (see Section 6.1). For example, Ag^+^ primarily disrupts proteins, membranes, and cellular functions, whereas Cu^2+^ promotes oxidative stress through redox cycling and damages essential biomolecules.^191^ Their antimicrobial effect can be tailored through nanoparticle architecture, with designs such as core–shell and Janus structures enabling control over metal distribution, ion release, surface reactivity, stability, and cytotoxicity.^191,193^ Other multimetallic nanostructures, such as Ag–ZnO hybrids and Au–Pt–Ag nanoparticles, have also demonstrated synergistic antimicrobial properties through combined metal or metal–oxide interactions.^16,177^

Although the multiple mechanisms of action exhibited by single-metal, bimetallic, and multimetallic MBNPs may reduce the likelihood of antimicrobial resistance development, they can also contribute to additional toxicity concerns (see Section 6.1).^17,195^ Moreover, distinguishing nanoparticle-specific antimicrobial and toxicity effects from those mediated by dissolved metal species remains a critical challenge. Therefore, appropriate experimental controls, including ion release and dissolution measurements under biologically relevant conditions, ROS-scavenging experiments, and light/dark controls for photocatalytic systems, are essential to determine the relative contribution of nanoparticle-mediated and dissolved-species-mediated effects.

For systemic applications, the use of MBNPs presents substantial challenges, including limited therapeutic efficacy, a potentially narrow therapeutic window, the need for high material doses, and significant toxicity when used for treating bacterial infections.^172^ Therefore, at this moment, their application is mostly confined to coating-based materials for wound dressings and implant devices.^17,192^ To improve their antimicrobial performance and facilitate systemic use, it is crucial to develop functional and stable nanostructures that exhibit lower biological toxicity with enhanced targeted bactericidal properties. In coating form, however, MBNPs show promising anti-biofilm properties, although their exact mode of action remains unclear.^16,196,197^ Considering these antimicrobial and anti-biofilm properties, along with their lower resistance susceptibility compared to traditional antibiotics, MBNPs are increasingly regarded as attractive candidates for coating applications.^16,172^ Despite encouraging *in vitro* safety profiles, comprehensive toxicological studies are still needed before MBNPs (in coating form) can be widely used as viable antimicrobial alternatives.^16,17,177^ Additionally, the potential emergence of novel resistance pathways necessitates systematic evaluation of the evolutionary pressure imposed by these materials relative to conventional antibiotics. Such assessments will be critical for determining their long-term sustainability as antimicrobial strategies (see discussion in Section 6.1).

### 4.2 Metal-Dependent Siderophore-Based Antibiotics and Other Metal Complexes

Iron plays a vital role in bacterial metabolism, growth, and replication.^174,198^ Iron acquisition is often regulated by the release of siderophore molecules that scavenge iron from its surroundings with high affinity.^198^ These iron–siderophore complexes are recognized by bacterial uptake systems and transported across the membrane, primarily via TonB-dependent transporters in Gram-negative bacteria and ATP-binding cassette (ABC) transporters in many Gram-positive bacteria^199,200^ (Figure 6). Taking advantage of these transporters, so-called “Trojan-Horse” antibiotics have been developed, in which siderophore–antibiotic conjugates are designed to improve uptake of antibiotics into bacterial cells, thereby significantly increasing antibiotic activity.^174,201^ The siderophore moiety enables recognition by bacterial iron-uptake pathways, facilitating transport of the conjugated antibiotic into the bacterial cell, executing its antibiotic effect^174,198,199^ (Figure 6). Based on this approach, in 2019, a novel antibiotic, cefiderocol,^174^ came to market. The structure consists of a catechol (a siderophore) covalently linked to ceftazidime, a cephalosporin (β-lactam) antibiotic, and is recognized and transported by TonB-dependent transporters into Gram-negative bacteria.^174,202^

Most of the Trojan-Horse antibiotic developments have been focusing on covalently linking β-lactams to siderophores,^174,203,204^ although examples of fluoroquinolones,^205,206^ lipopeptides,^207^ and other traditional antibiotics have also been reported.^201^ Other approaches of siderophore-based antibiotics involve using metal ions like gallium (Ga) and bismuth (Bi) instead of iron, which, due to their similar valence state of +3, geometry, and similar coordination, can mimic and utilize iron’s transport mechanisms.^208–210^ Gallium citrate is one such metal-based antimicrobial agent currently in clinical trials as a target for *P. aeruginosa* infections.^63,211^ Gallium ions are actively transported into bacterial cells, and once inside the bacterial cell, gallium has been shown to disrupt key biological processes by interfering with iron-dependent enzymes and disrupting iron homeostasis, leading to disruption of cellular functions and eventual bacterial death^209–211^ (Figure 6). Recently, bismuth-based antibiotics have gained attention for their potential to combat *P. aeruginosa* and inhibit biofilm formation.^212^ Bismuth compounds have a long history of use in medicine, particularly in the treatment of *Helicobacter pylori* infections.^212,213^ However, their antibiotic activity is currently being expanded to other bacterial species, mostly in combination therapies with other antibiotics.^212^ Bismuth can work as an antibiotic in nanoparticle form (as an MBNP), as ions (as a siderophore-based antibiotic), or as bismuth thiol (sulfur-containing group) complexes, which enhance lipophilicity and facilitate bacterial membrane penetration, potentially increasing its efficacy.^212–214^

Siderophore-based antibiotics and metal complexes, including gallium-based and bismuth thiol compounds, represent promising approach to tackling AMR (Figure 6). However, bacterial resistance mechanisms can significantly hinder the effectiveness of siderophore–antibiotic conjugates. In *P. aeruginosa*, adaptive resistance has already been reported through the blockage of siderophore-mediated uptake.^215^ Similarly, *E. coli* and other Enterobacteriaceae can develop resistance to catechol-based conjugates by modifications in receptor expression.^198^ If this resistance can be mitigated, continued research may enable siderophore and metal complex antibiotics to offer additional therapeutic options in the future.

### 4.3 Carbon-Based Nanoparticles

Carbon-based nanoparticles (CBNPs) are an emerging antimicrobial strategy with significant potential.^18,19,216,217^ Examples of carbon-based nanoparticles (CBNPs) include fullerenes, nanodiamonds, carbon nanotubes (CNTs), graphene oxide (GO), and carbon dots (CDs). Among these, GO and CDs have recently attracted the greatest attention for antimicrobial applications due to their tunable surface chemistry, multifunctionality, and comparatively favorable biocompatibility profiles.^18,169,170,218,219^ Additionally, CBNPs have been shown to be conjugated with conventional antibiotics or antimicrobial peptides for synergistic antimicrobial therapies.^220–222^ The antimicrobial activity of CBNPs is strongly dependent on their physicochemical properties, including composition, morphology, and surface chemistry.

Similar to MBNPs, CBNPs also kill bacteria by inducing oxidative stress, especially when functionalized with oxygen-containing groups, such as hydroxyl and carboxyl^170,173,223^ (Figure 6). Additionally, some CBNPs also exert an antimicrobial effect through physical or mechanical disruption, often described as “physical puncture”. This mechanism is especially relevant for graphene-based CBNPs and some CNTs, whose sharp edges, nanoscale defects, and high aspect-ratio structures can physically interact with bacterial membranes, causing membrane deformation, lipid extraction, and loss of membrane integrity.^18,170,224,225^ Furthermore, CBNPs, specifically graphene-based materials and CDs,^226^ were found to have significant efficacy in disrupting biofilms through bacterial adhesion inhibition and disrupting biofilm maturation.^18,227^

Among CBNPs, CDs have emerged as a particularly promising subgroup, including graphene quantum dots (GQDs), carbon quantum dots (CQDs), and carbon polymer dots (CPDs).^222,225^ Unlike larger carbon nanomaterials, CDs combine nanoscale dimensions, high aqueous dispersibility, and versatile surface chemistry, enabling their use both as antimicrobial agents and as delivery platforms for antimicrobial compounds.^225^ Surface functionalization with cationic groups can increase electrostatic interactions with negatively charged bacterial membranes, facilitating bacterial targeting and membrane disruption. Additionally, incorporation of photosensitizers enables photodynamic antimicrobial effects, while surface engineering can provide photothermal antibacterial functionality.

Graphene-based materials can also serve as photothermal or photodynamic antimicrobial platforms.^225^ Photothermal materials convert near-infrared irradiation into localized heat, causing thermal membrane disruption, whereas photosensitized CBNPs result in increased ROS generation. In addition, graphene-based materials and functionalized CDs can interfere with bacterial adhesion, EPS formation, and quorum sensing, thereby reducing biofilm development and virulence-associated behavior.^219,227^

CBNPs have been shown to possess strong antibacterial properties against a wide variety of Gram-negative and Gram-positive bacteria, depending on their functionalization.^18,227^ The antimicrobial performance of CBNPs mostly depends on the design and synthesis, including the length, size, composition, surface area, purity, and surface functionalization.^18,228^

The current application of CBNPs remains, however, largely focused on non-systemic approaches, such as material coatings, due to challenges associated with toxicity, biodistribution, and long-term safety.^173^ In particular, the relationship between surface chemistry, ROS generation, antimicrobial selectivity, and cytotoxicity requires systematic evaluation, as factors such as dosage, treatment time, and bacterial species can significantly influence the antimicrobial efficacy and safety profile of CBNPs.^18,170^ Further studies are also required to understand how CBNP properties influence activity within complex biofilm environments. Therefore, rational design of CBNPs, particularly CDs, will be essential for balancing antimicrobial potency, biofilm efficacy, and biological safety to facilitate their translation into biomedical applications.^18,170,229^

### 4.4 Silicon-Based Antimicrobial Drug Delivery Systems

Silicon-based scaffolds, particularly mesoporous silica nanoparticles (MSNs) and zeolites, show great promise for antimicrobial drug delivery.^230^ Although reports on these silicon-based antimicrobial drug delivery systems are relatively sparse, their potential is high due to their high versatility and tunability. MSNs feature highly ordered pore structures with diameters ranging from 2 to 50 nm. MSNs are typically synthesized by templating cetyltrimethylammonium bromide, around which a silica network forms via hydrolysis and condensation of tetraethyl orthosilicate. To enhance the colloidal stability, the surface of the MSN particles is often functionalized and/or capped, typically with polymers, lipid coatings, or metal ions and metal nanoparticles.^230–232^ They can encapsulate substantial amounts of drug molecules, protect their load from environmental degradation, and enable sustained and controlled release^175^ (Figure 6). Their high surface area, high chemical and thermal stability, modifiable surface chemistry, large pore volumes, and biocompatibility make them attractive for drug delivery applications.^175,230^ In addition, MSNs can be engineered to respond to specific environmental stimuli such as pH, ionic strength, or enzymatic activity, allowing for improved targeted release at infection sites while minimizing side effects like off-target exposure and toxicity.^233^ Antimicrobial agents are most often loaded/adsorbed in the pores,^175,234–236^ or conjugated onto the surface of MSNs.^230,233,234,237^ The loading and conjugation of numerous different antimicrobial components, including AMPs and conventional antibiotics,^238^ but also metal ions and MBNPs, have been investigated, focusing on, *e.g.*, systemic therapies,^175,234^ targeting of biofilms,^175^ metal-antibiotic synergistic approaches,^230,239^ and implant coatings.^237^ Despite encouraging *in vitro* results, clinical translation of MSNs is still hindered by limited *in vivo* evaluations and unresolved challenges related to blood circulation, clearance, metabolism, and long-term safety.^230^ Although silica-based materials are generally recognized as safe by the FDA, concerns about long-term toxicity, limited targeting, and trace metal accumulation from MSNs, as well as for zeolites, present significant challenges for their biomedical application.

Zeolites are hydrated aluminosilicates composed of interconnected SiO_4_^4–^ and AlO_4_^5–^ tetrahedral units.^176^ These tetrahedra form a three-dimensional crystalline framework with well-defined channels and cavities, the dimensions of which vary depending on the specific arrangement of the silicon and aluminum units. The resulting porous network can host various molecules and cations, including metal-based antimicrobial compounds,^240^ making zeolites suitable for ion exchange and drug encapsulation applications. Zeolites have only very recently been proposed as a drug delivery system for antibiotics.^176^ Although not on the nanoscale, Domke et al. (2024) studied a Mg^2+^, Ca^2+^, Sr^2+^, and Zn^2+^ exchanged X-type zeolite carrier for ciprofloxacin, showing promising adsorption properties with adequate antimicrobial activity and release.^176^ While the use of zeolites as antibiotic carriers is a recent development in pharmaceutical applications, their capacity to adsorb antibiotics has been previously investigated for wastewater purification.^241,242^ In this new form as a potential antimicrobial material, antimicrobial zeolites might find applications in bone-scaffold fillers and implant coatings.^176^

## 5 Supramolecular Antimicrobial Nanoparticles and Drug Delivery Strategies

To enhance the efficacy and properties of both clinically approved and newly developed antimicrobial compounds, research efforts have increasingly focused on supramolecular antimicrobial nanoparticle systems.^19,243–245^ Supramolecular chemistry, literally meaning “beyond the molecule”, is the study of molecular systems formed from multiple components that associate through non-covalent interactions.^246^ A key concept within this field is molecular self-assembly, in which these non-covalent interactions, such as hydrogen bonding, π–π stacking, electrostatic forces, van der Waals interactions, and hydrophobic effects, drive the *spontaneous and reversible* organization of molecules into well-defined, functional structures.^247,248^ Furthermore, the assembly of two or more distinct components through complementary non-covalent interactions is referred to as co-assembly. In contrast, if the supramolecular assembly requires external energy input or direction, we will refer to this process as “*driven assembly*”.^249^

Depending on the intrinsic chemical properties of antimicrobial compounds, some antimicrobials show single-component self-assembly behavior (Figure 7). Both natural and synthetic antimicrobials have been reported to self-assemble into various structures, including micelles, nanofibers, and nanosheets, among others.^31,250^ Other antimicrobial compounds can, instead of self-assembling, be incorporated into supramolecular assemblies formed from synthetic polymers, biomolecules, or inorganic NPs (Figure 7). Common approaches for such integration include encapsulation, adsorption, entrapment, and co-assembly strategies. A wide range of supramolecular drug delivery vehicles, including polymer-based, lipid-based, and hybrid systems, have been investigated for their potential in targeted antimicrobial delivery^19,20,64,244,245,251^ (Figure 7). These supramolecular antimicrobial nanoparticles are engineered with the primary goals of minimizing the risk of AMR development and lowering the side effects of the antimicrobial agents, *e.g.*, cytotoxicity or adverse immune responses.^252,253^ Additionally, they aim to improve the effectiveness and activity. Specifically, such nanoparticles improve critical aspects of the loaded antimicrobial agent, including protection from degradation, increased stability, enhanced bioavailability, targeted adhesion, and precise delivery to the active site.^33^ In the following subsections, we will highlight recent advances and innovations in the field of supramolecular antimicrobial nanoparticles for future systemic and non-systemic therapeutic antimicrobial applications.

**Figure 7:**
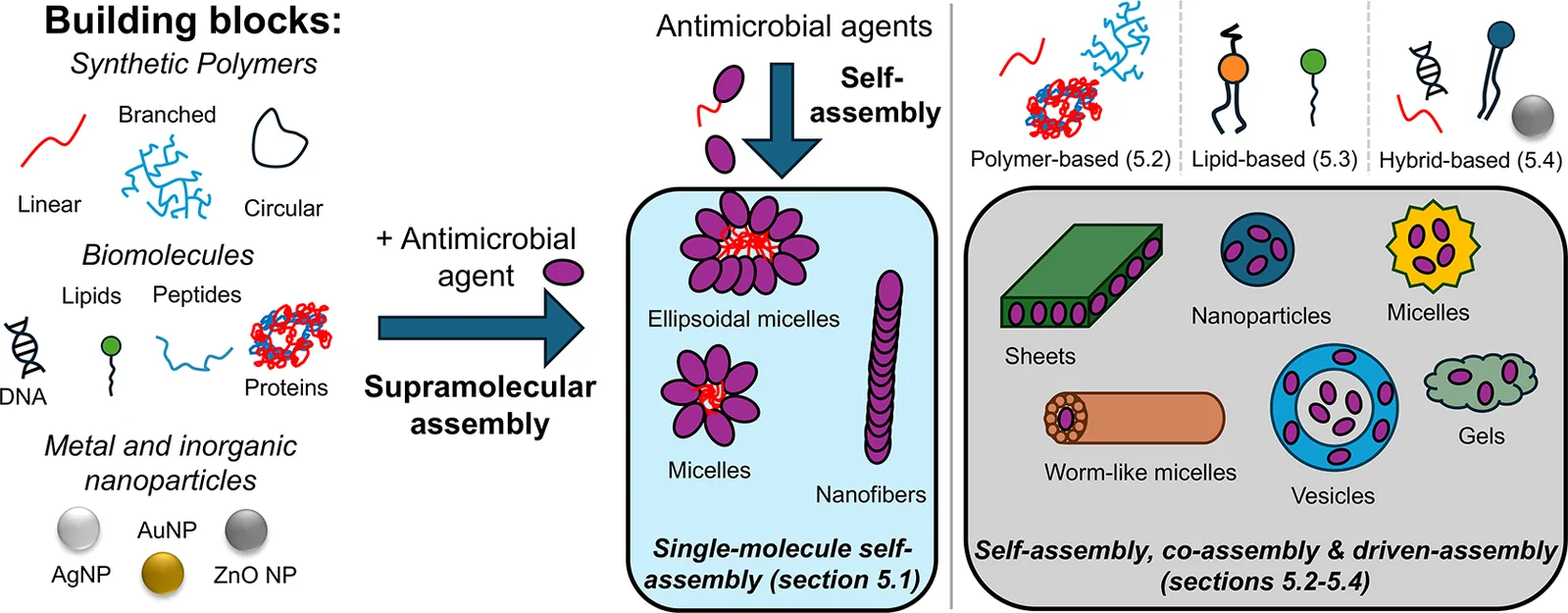
Overview of supramolecular assembly strategies used to construct antimicrobial drug delivery systems. Recent advances in self-assembled, co-assembled, and driven-assembled systems aimed at antimicrobial delivery are discussed. In some cases, antimicrobial compounds themselves are capable of self-assembly into structures such as micelles, ellipsoidal micelles, or nanofibers, as discussed in Section 5.1. Building blocks such as synthetic polymers, biomolecules, and metal or inorganic nanoparticles can assemble into a wide range of nanostructures, including sheets, nanoparticles, micelles, worm-like micelles, vesicles, and gels, that can serve as carriers for antimicrobial agents (Sections 5.2, 5.3, and 5.4).

### 5.1 Single-Component Self-Assembling Antimicrobials

The formation of supramolecular assemblies can enhance the therapeutic efficacy of small antimicrobial compounds by improving their stability and enabling controlled release.^254^ Through careful design, these compounds can self-assemble into supramolecular structures ranging from a few to thousands of molecules at a wide range of compositions, morphologies, and antimicrobial loadings.^254,255^ Such assemblies not only allow for a gradual and targeted release of antimicrobial agents but can also contribute to minimizing side effects and improving stability.^250^ Supramolecular structures formed by AMPs have already demonstrated increased antimicrobial activity, reduced hemolytic response, reduced cytotoxicity, and enhanced resistance to degradation by serum proteins compared to non-assembling counterparts.^250,256,257^

Self-assembling antimicrobials of natural origin are scarce. Examples include peptide structures belonging to the class of AMPs, such as human α-defensins,^258^ yeast-derived EAK16,^259^ and the lipopeptide daptomycin.^260,261^ Additionally, colistin had previously been suggested to self-assemble into ordered aggregates, although this was recently disproven.^83^ Due to the improved properties that self-assembling antimicrobial compounds can exhibit and the lack of natural self-assembling antimicrobials, research efforts have been made in the development of (*de novo* designed) synthetic AMPs and mimics with self-assembly properties.^31,126,239,256,257^ Next to self-assembling synthetic antimicrobial peptides,^250,255^ also peptoids,^31^ and polymers^257^ have been explored preclinically as antibiotic candidates. For example, Xu et al. (2014) explored synthetic nanofiber-forming antimicrobial peptides and observed that, in certain cases, the formation of these assemblies led to an improved balance between antimicrobial efficacy and cytotoxicity.^255^ Nielsen et al. (2022) evaluated a broad range of self-assembling peptoids as potential therapeutics against the so-called ESKAPE pathogens.^31^ Depending on the sequence, some peptoids assembled into helical bundles, while others formed core–shell ellipsoidal or worm-like micelles, displaying widely varying antimicrobial and antibiofilm activities, depending on their self-assembly behavior.

In the search for self-assembling antimicrobials, in addition to synthetic antimicrobial peptides and mimics, the conjugation of functional groups to classical and engineered antibiotics has been investigated.^256,262^ For example, antibiotics have been covalently linked to lipids, nucleic acids, photosensitizers, polymers, or metal nanoparticles, resulting in self-assembling properties.^30,262,263^ Conjugation affects both the hydrophobicity and amphiphilicity of the molecules, thereby promoting self-assembly through a balance of entropic contributions (mainly from hydrophobic effects) and enthalpic interactions (such as hydrogen bonding and electrostatic interactions). Examples have been shown of the conjugation of traditional antibiotics, such as penicillin,^264^ fluoroquinolones,^265^ and aminoglycosides,^262^ resulting in single-component supramolecular assemblies, such as micelles and nanofibers. In addition, self-assembling AMP-conjugates have been designed and developed.^256,266^ These assemblies were subsequently compared with their non-conjugated counterparts to assess the impact of conjugation on antimicrobial activity. Several studies reported that, in addition to changes in activity and stability, conjugation could also alter the mode of action by modifying membrane affinities.^256,266–268^

Due to changes in chemical properties, membrane activity and mode of action can be influenced.^250,266,268,269^ For example, Chu-Kung et al. (2010) found that conjugating the AMP AKK with a fatty acid enhanced its membrane affinity.^268^ However, in a micellar state, at concentrations above the critical aggregation/micelle concentration (CAC/CMC), the assemblies did not show any antimicrobial effect. In other cases, self-assembly generates supramolecular antimicrobial architectures with enhanced activity compared with their individual molecular components, highlighting the role of assembly in regulating bacterial membrane interactions.^270^ Generally, the CAC/CMC is a crucial parameter for characterizing the self-assembly behavior of amphiphilic antimicrobial structures. These findings highlight the need to balance hydrophobic modifications and self-assembly to optimize antimicrobial performance.^266,268,269,271^

Notably, there is often a significant mismatch between the CAC/CMC and the minimal inhibitory concentration (MIC). In many cases, CAC/CMC values are orders of magnitude higher than MICs, indicating that self-assembly is unlikely to play a role under therapeutic conditions.^83^ For self-assembly to contribute meaningfully to antimicrobial function, the CAC should ideally overlap with the MIC, enabling efficient drug release within a physiologically relevant concentration range. Alternatively, there should be a trigger mechanism of ordered aggregate disintegration based on external factors, such as changes in pH, ionic strength, enzymatic activity, or membrane interactions.

Furthermore, the resulting supramolecular morphology can play a critical role in determining biological activity. Comparing assemblies formed from identical or closely related building blocks has demonstrated that spherical micelles, worm-like micelles, and nanofibers can exhibit substantially different antimicrobial properties.^31,272,273^ These differences can arise from variations in surface curvature, aspect ratio, molecular packing, and accessibility of functional groups. Highly elongated assemblies may provide extended contact surfaces that promote interactions with bacterial membranes, whereas more spherical micelles can present a high local density of antimicrobial motifs due to their increased curvature.^272–274^ Hamley et al. (2025) recently demonstrated that C16-XKK lipopeptides, which formed worm-like micelles/fibrils, exhibited higher antimicrobial activity than C16-KXK lipopeptides forming planar bilayer structures.^273^ However, increased aspect ratio alone does not universally correlate with enhanced antimicrobial activity, as morphology-dependent effects are strongly influenced by additional factors, including protease protection, molecular organization, and the physicochemical properties of the assembled system.^275^ For example, a study by König et al. (2023),^276^ revealed that minor modification in the sequence of the first amino acids in a peptide-forming nanosheets significantly altered the enzymatic stability, possibly by modulation of cohesive energy and the physical lifetime of the assembly.^255,276^ Further systematic studies are therefore needed to clarify how supramolecular morphology and structural stability regulate antimicrobial performance.

The development of single-component self-assembling antimicrobial compounds remains challenging, largely because their properties, such as assembly behavior, stability, and antimicrobial activity, are difficult to predict and control.^266,268^ Moreover, the development of self-assembling conjugates or entirely synthetic antimicrobials entails lengthy and costly drug development processes, particularly when new chemical entities are involved. Despite these challenges, self-assembling antimicrobials hold promise for future antimicrobial therapeutics if their assembly and antimicrobial performance can be optimized. As *in vitro* studies often fail to predict *in vivo* outcomes, *in vivo* studies are essential in this antimicrobial discovery process. Therefore, systematic investigation of their morphology-function relationships, stability, circulation time, accumulation at the target site, and metabolic fate is essential before they can be used clinically.^256^

### 5.2 Polymer-Based Antimicrobial Nanoparticles

Polymers offer high versatility for selective drug release due to their wide-ranging and tunable chemical properties. They can be derived from natural sources (biopolymers) or synthetically designed with tailored features and diverse configurations (see Section 3.1.2). Biopolymers, including (poly)­peptides/proteins and saccharides, serve as polymer classes that are often biocompatible, making them attractive for biomedical applications. The versatility of polymer-based materials is especially visible in the development of synthetic “smart-material” strategies such as surface-charge switching polymers and acid-degradable acetal/ketal functionalities, which have already been employed for targeted, pH-responsive antibiotic delivery.^13,185^ Their structural tunability enables a broad range of antimicrobial delivery strategies, as illustrated in Figure 8 and further discussed in the following section.

**Figure 8:**
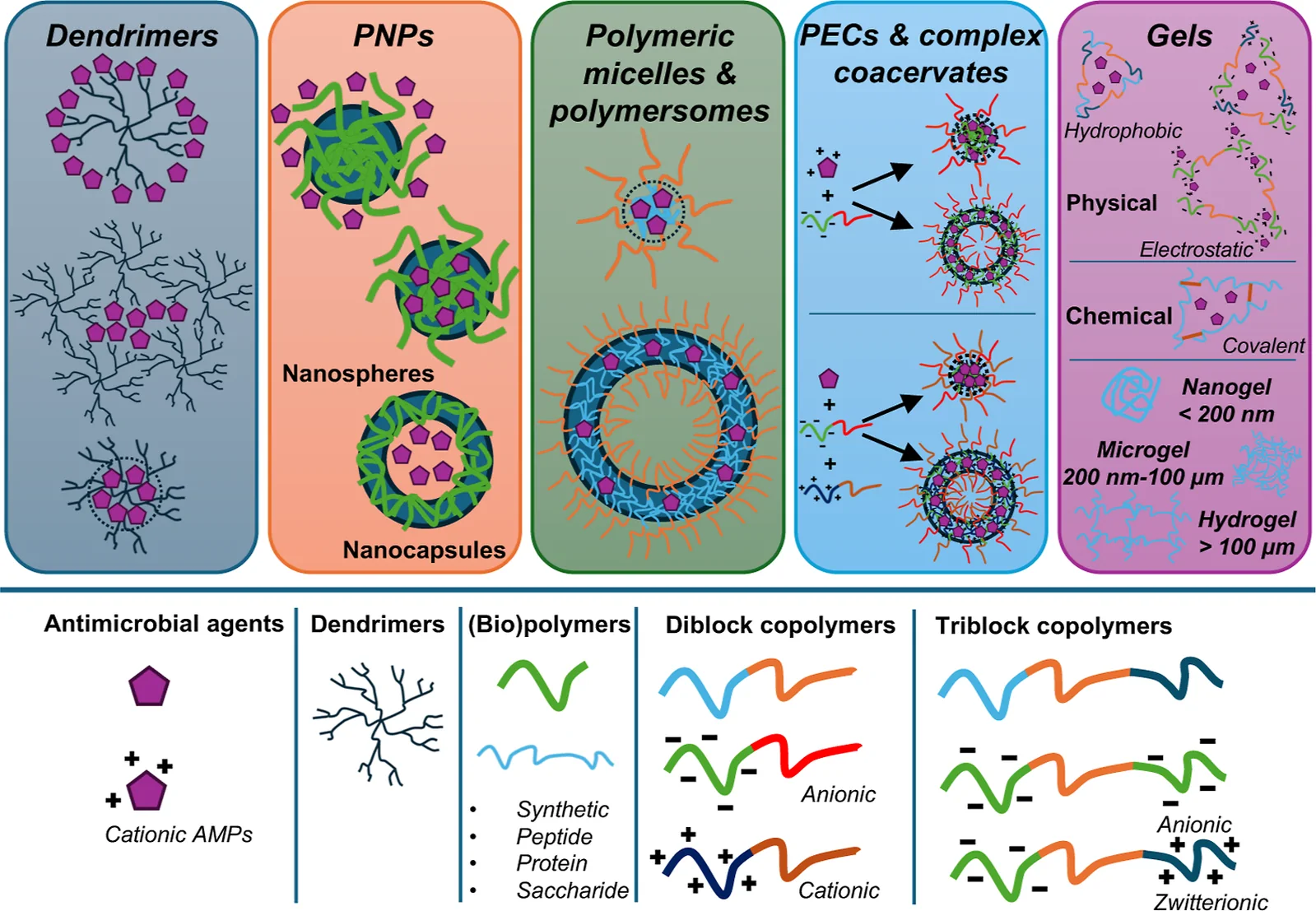
Overview of polymer-based antimicrobial delivery systems based on supramolecular assembly. Scaffolds meant for antimicrobial delivery include dendrimers, polymeric nanoparticles, polymer micelles, polymersomes, polyelectrolyte complexes, complex coacervates, and gels. Dendrimers can adsorb, encapsulate, or entrap antimicrobial agents. PNPs formed through driven-assembly methodology form nanospheres or nanocapsules, where nanospheres adsorb or entrap agents, while nanocapsules encapsulate them. Diblock copolymers can self-assemble into micelles and polymersomes (hydrophobic interactions) or, if charged (electrostatic interactions), into complex coacervate core micelles/vesicles (C3Ms/C3Vs), which can encapsulate agents or incorporate them as part of the structure. Gels, in the form of nanogels, microgels, and hydrogels, can be formed through physical (hydrophobic interactions or electrostatic interactions) or chemical crosslinking and can be loaded with antimicrobial agents. In this section, we discuss recent advances in these polymer-based systems for antimicrobial delivery.

#### 5.2.1 Dendrimers

Dendrimers (from the Greek word for tree, dendra) are hyper-branched polymers featuring a central core from which monomeric branches extend, forming globular structures.^155^ Each branching level, also known as a generation, increases the number of functional groups available for conjugation.^155,277^ Due to their advantageous characteristics, including high water solubility, low immunogenicity, biocompatibility, precise structural control, well-defined molecular weights, and tunable surface functionalities, dendrimers have attracted considerable interest for various biomedical applications.^244,277,278^ Moreover, poly­(amidoamine) (PAMAM) dendrimers are among the most potent and extensively studied, although their cytotoxicity remains a key challenge in their therapeutic application.^279^ As previously shown in Figure 3 and discussed in Section 3.1.2, dendrimers can also be designed as AMP mimics,^280,281^ and thereby exhibit antimicrobial activity themselves, which also have been shown to synergize with conventional antibiotics.^282–284^

Compared to AMP-mimicking dendrimers, the literature on the use of dendrimers as a drug delivery platform for antibiotics or antibiotic enhancers is relatively sparse.^243,244,279,285^ As the most studied dendrimer, PAMAMs have also been investigated and shown to be a useful scaffold for the delivery of antibiotics.^286,287^ These studies primarily focus on extending sustained antibiotic release by forming nanostructures such as conjugates and supramolecular assemblies.^244,288,289^ Antibiotics are then (reversibly) covalently linked or non-covalently entrapped, encapsulated, or associated with dendrimers, respectively^277,286,290^ (Figure 8). For example, Chosy et al. (2024) recently reported the development of vancomycin-polyguanidino dendrimer conjugates, which demonstrated a significant improvement in efficacy against both Gram-positive and Gram-negative bacteria compared to vancomycin alone.^288^ These antibiotic conjugates showed no acute toxicity toward mammalian cells or hemolytic activity and were effective in eradicating biofilm-forming Methicillin-resistant *S. aureus* (MRSA). Yesbeck et al. (2024) used amine-terminated PAMAM dendrimers to create a degradable, low-cytotoxic dendrimer hydrogel for successful cefazolin treatment in bone grafting.^287^ Other dendrimeric supramolecular assemblies, such as vesicles, have also been explored for antibiotic delivery.^289^ Omolo et al. (2018) synthesized vesicle-forming poly­(ester-amine) dendrimers in combination with a diblock copolymer of methoxy poly­(ethylene glycol)-*b*-poly­(ε-caprolactone) (mPEG-*b*-PCL).^289^ They successfully loaded these dendrimers with vancomycin and demonstrated sustained release profiles. Dendrimers can also act as templates for antimicrobial metal nanoparticles, offering precise control over particle size and structure.^291,292^ This high versatility of dendrimers allows for heterofunctionalization, facilitating the development of antimicrobial combination therapies on the same dendrimer assemblies.^291,292^

#### 5.2.2 Polymeric Nanoparticles

Polymeric nanoparticles (PNPs), ranging from 10 to 500 nm, consist of biocompatible polymeric matrices in which a therapeutic agent can be entrapped, adsorbed, or covalently bound.^293^ Natural biopolymers, as well as synthetic polymers, are widely used to form PNPs with varying properties.^294^ PNPs represent a typical example of driven assembly, where external forces/processes are required to induce nanoparticle formation, *e.g.*, by emulsion-solvent evaporation, (flash) nanoprecipitation, or dialysis.^27,295,296^ PNPs can be broadly classified into nanospheres and nanocapsules based on their internal architecture^293,297,298^ (Figure 8). Nanospheres, often prepared through emulsion-solvent evaporation or nanoprecipitation, feature a solid, homogeneous polymer matrix in which drug molecules are either embedded throughout or adsorbed onto the surface.^293,298^ In contrast, nanocapsules, often prepared through nanoprecipitation or dialysis, possess a core–shell structure, where the active compound is confined within a central cavity. This cavity is typically liquid and surrounded by a polymeric membrane^298^ (Figure 8).

Polymeric nanoparticles have been extensively studied for applications involving controlled antibiotic release.^27,296,297^ Over the past decade, numerous polymeric nanoparticle systems with varied antimicrobial loads have been developed.^27,295,299–302^ Although many studies have been published, most focus on nanoparticle characterization rather than antimicrobial activity or release profiles. Therefore, it is often unclear how effective the proposed antibiotic formulation is compared to its non-embedded counterpart. Although often overlooked, some antibiotic PNPs have reported improved antibiotic efficacy^27,297,299^ and shown effective eradication of biofilms.^300^ Beyond composition and loading efficiency, nanoparticle morphology has also been shown to influence antimicrobial performance through effects on bacterial interactions, stability, and drug accessibility, where smaller and less elongated PNPs were found more effective.^303^

Many PNP antimicrobial delivery systems primarily focus on natural biopolymers such as chitosan (polysaccharide),^304,305^ cyclodextrins (oligosaccharide),^306^ gelatin (protein),^301^ and polysaccharides,^299,307^ as well as synthetic, but biocompatible polymers like poly­(lactic-*co*-glycolic acid) (PLGA)^295,302^ and polycaprolactone (PCL).^308^ These polymers have been shown to effectively encapsulate, adsorb, or entrap a broad spectrum of antimicrobial agents, including bacteriophages,^309^ antimicrobial mAbs,^310^ AMPs,^239,295,311^ glycopeptides,^296^ fluoroquinolones,^312^ aminoglycosides,^304,313^ and β-lactams,^308^ among many others. Antimicrobial-loaded PNPs, owing to their biocompatibility, versatility, and strong research foundation, represent a highly promising platform for future therapeutic applications, provided they can be successfully translated and evaluated for therapeutic properties *in vivo*.

#### 5.2.3 Polymeric Micelles and Polymersomes

Polymeric micelles and polymersomes are usually prepared from (most often synthetic) amphiphilic block copolymers. These block copolymers contain a coupling of a hydrophobic block with a neutral hydrophilic block, usually poly­(ethylene oxide) (PEO), although alternatives are emerging in drug delivery design.^314^ Hydrophobic interactions drive the self-assembly of micellar and vesicular structures, after which the hydrophilic blocks stabilize the structures. This self-assembly is dependent on the polymer architecture (diblock, triblock, and block lengths) and desired drug-loading.^315^ Moreover, their resulting morphology is mostly related to the block lengths of the hydrophobic blocks, where shorter blocks tend to form micelles instead of vesicles. In polymeric micelles, the hydrophobic blocks form a core, while the hydrophilic block forms a shell^316^ (Figure 8). For vesicular morphologies, also known as polymersomes, which were first proposed as a drug delivery vehicle in the 1990s, the relatively larger hydrophobic blocks can form a vesicular bilayer.^315,316^ This bilayer then encapsulates an aqueous cavity, with the hydrophilic blocks pointing into the lumen of the vesicle and outwards^317^ (Figure 8). Due to the amphiphilicity of the polymer blocks, and therefore, also in the structures, antimicrobial agents or drugs can be carried in either the hydrophilic parts or the hydrophobic bilayer, depending on the desired antimicrobial load. The antimicrobial agent can either be physically encapsulated^296,318^ or chemically linked to the polymer (conjugated).^319,320^

Although less extensively studied than polymeric nanoparticles (PNPs), polymeric micelles and polymersomes loaded with antimicrobial agents, ranging from traditional antibiotics to AMPs and metal nanoparticles, have received considerable research attention in recent years.^318,321–326^ A wide range of polymer types and antimicrobial cargoes have been explored in the last decade. These include diblock copolymers,^318,321,322^ triblock copolymers, often Pluronics,^323–325^ as well as peptide–polymer hybrids,^325^ inclusion-complex cyclodextrin-antibiotics,^327^ and polymer–antibiotic conjugates.^319,320,325^ Systems incorporating charged, hydrophilic blocks with pH-responsiveness have also been investigated for enhanced release in acidic environments.^326,328^ A significant portion of the literature in this field emphasizes biofilm eradication as a key therapeutic target.^323,325,326^ Continued research into optimizing polymer architectures, having control over antibiotic release, and understanding interactions with bacterial biofilms will be critical for translating these nanoparticles into clinically viable treatments.

#### 5.2.4 Polyelectrolyte Complex and Complex Coacervate Assemblies

As an alternative to self-assembled micelles based on hydrophobic interactions, electrostatically driven polymer-based nanostructures have gained significant research interest since work in the late 1990s.^329–331^ These so-called polyelectrolyte complexes (PEC), in the literature also sometimes referred to as polyion complexes (PIC), interpolyelectrolyte complexes (IPEC), and block ionomer complexes (BIC), are assemblies based on the mixing of polymeric components with opposite charge.^329–332^ When these assemblies show liquid-like behavior in their core, *i.e.*, the polyelectrolyte chains remain dynamically mobile, allowing diffusion, reorganization, or even exchange, they are often coined complex coacervate core micelles (C3Ms).^332^ Analogous to polymeric micelles, these complexes are prepared from one or more block copolymer species. Block copolymers used for micellization consist of a charged block and a neutral block (often PEO), which undergo complexation with another oppositely charged block copolymer, homopolymer, or biopolymer (Figure 8). The oppositely charged polyelectrolytes induce a liquid–liquid phase separation into a coacervate (dense) phase and a dilute phase. The coacervates consist of a core (formed by enthalpic contributions from electrostatic attractions), while the neutral hydrophilic block(s) of the polymer(s) form(s) a protective shell around the highly charged core.^332^ The hydrophilic block ensures effective entropic stabilization, enhancing its colloidal stability. These C3Ms typically exhibit a core–shell morphology, with their size influenced by various factors such as the polyelectrolyte block length, ionic strength, charge density, and the number of polyelectrolyte moieties.^332–334^ Depending on the polymer properties, other morphologies of these supramolecular assemblies can be formed, such as worm-like micelles and vesicles (termed C3Vs), although micellar complexes are most common.^335,336^

Over the past decade, several studies have reported antibiotics being entrapped within the polyelectrolyte core,^317,337,338^ or, in the case of charged antibiotics such as AMPs, incorporated directly into the structure through co-assembly^29,317,336,339–342^ (Figure 8). Additionally, although not yet demonstrated, for C3Vs, antimicrobial agents can also be loaded in the aqueous lumen of the vesicle.^343^ These C3M and C3V systems offer several advantages over multiple other drug delivery platforms: they are relatively simple to prepare, do not require stabilizing agents or harsh chemicals, and have shown improved stability, retention, and preservation of antimicrobial activity.^29,344,345^ Despite these benefits, several challenges remain.^345^ One major issue is their sensitivity to ionic strength, which can weaken electrostatic interactions, which is particularly problematic for C3Vs, which are destabilized even at physiological salt concentrations.^332,336,346–348^ Additionally, the CMCs/CACs of these systems are often much higher than the MIC values.^29,332^ Finally, a balance must be achieved between ensuring colloidal stability during transport and allowing for controlled disassembly at the target site. Ultimately, successful upscaling and clinical studies will be crucial for translating these systems into viable topical and systemic therapeutic applications.

#### 5.2.5 Nanogels, Microgels, and Hydrogels

In the last major category of polymer-based antimicrobial delivery platforms, we discuss the gel formation of polymer materials into nanogels, microgels, and hydrogels. A gel is a three-dimensional network of crosslinked polymer chains capable of retaining a large amount of water within its structure.^349,350^ This gelation process can occur on different length scales: at the nanoscale, forming nanogel particles (<200 nm), at the microscale, microgels (200 nm −100 μm), or at the macroscale, forming hydrogels (>100 μm) (Figure 8). Central to gel formation is the presence of a crosslinking mechanism, resulting in drug delivery potential and water-retaining capacity.^349,351,352^ Chemical crosslinking typically involves covalent bonding, such as through polymer coupling reactions or the oxidation of thiol groups to form disulfide bridges. Physical crosslinking, often based on self-assembly, can occur due to hydrophobic interactions, similar to polymeric micelles and polymersomes, but also hydrogen bonding and electrostatic interactions, similar as in C3Ms and C3Vs. Gels can be formed from a wide variety of polymers, including homopolymers, diblock or triblock copolymers, and natural biopolymers (including inclusion-complexing cyclodextrins^353^), provided they offer functional groups that enable chemical or physical crosslinking^352,353^ (Figure 8).

Nanogels are ideal for drug delivery because they are structurally stable, biocompatible, responsive to environmental changes via swelling or deswelling, and offer high drug-loading capacity compared to other polymer scaffolds.^349^ As they are on the nanoscale, they have systemic potential as particles. Microgels and hydrogels are more commonly used for localized or depot-based delivery, and their antimicrobial effect is dependent on release from the gels.^349,350^ Microgels represent a middle ground between nanogels and hydrogels, offering higher drug-loading capacity than nanogels while enabling faster drug release compared to hydrogels. Due to their intermediate size and responsiveness, they are particularly well-suited for applications such as subcutaneous drug delivery, facilitating localized, controlled release. The key strength of hydrogels over nanogels and microgels is their ability to sustain the release of high quantities of drugs over extended periods, which can improve therapeutic outcomes while minimizing systemic side effects.^349^ They can be tailored for infection-site targeting, enabling topical treatments in, for example, wound dressings, or parenteral administration for internal infections.^354^ While release from these systems can occur through simple diffusion, the versatility of polymer properties has also enabled the development of stimuli-responsive gels.^349,351^ Stimuli-responsiveness can be triggered by light, heat, pH, enzymatic activity, magnetism, or other environmental cues.^349^

A multitude of different antimicrobial agents have successfully been incorporated into nanogels,^355–358^ microgels,^345,351^ and hydrogels,^356^ opening new avenues for improving antimicrobial therapies.

The most common polymers explored for antimicrobial gel formulations include (derivatives of) chitosan,^359^ hyaluronic acid,^358,360^ alginate,^361^ carbopol (polyacrylic acid),^357^ among others. Conventional antibiotics, such as ciprofloxacin^356,360^ and tetracycline,^361^ and emerging antimicrobial agents, such as AMPs and mimics^238,351,358^ and metal-based nanoparticles,^183,362^ have been explored in gel-based delivery systems. More recently, gel-loaded carbon nanodots have also gained attention for their antimicrobial potential.^363^ Another recent early-stage innovation of an emerging non-traditional antimicrobial strategy is, for example, the use of ozone nanobubbles,^364^ loaded into hyaluronic acid nanogel carriers.^365^

These antimicrobial gel systems have been proposed for a variety of applications, including anti-biofilm treatments,^357^ antimicrobial coatings for implants,^359^ wound dressings,^356,366^ and injectable antimicrobial delivery platforms,^354,367^ highlighting the high versatility of this field.

While this field holds great promise, several challenges remain before gel-based antimicrobial delivery systems can reach clinical application. Gels often require transformation into a stable dry formulation, *e.g.*, via lyophilization, to ensure long-term storage and shelf-life.^351^ Moreover, their safety and therefore regulatory approval, drug release profiles, antimicrobial efficacy, and interactions within complex biological environments like wounds must be thoroughly evaluated.^349^ Further research is essential to understand how gel composition, formulation strategies, and upscaling processes impact the stability and compatibility of antimicrobial agents within these systems.

### 5.3 Lipid-Based Antimicrobial Nanoparticles

Lipid-based nanoparticles have been a subject in the field of drug delivery since the 1960s, when the structural organization of phospholipids into self-assembled liposomes was first elucidated^368,369^ (Figure 9). Later, the field developed further with effective liposomal drug loading and surface modifications to improve the functional properties of liposomes.^369^ Following technological advances in high-pressure homogenization and high-pressure mixing, in the 1980s and 1990s, other lipid-based nanoparticles (often based on driven assembly) could be developed, such as nanoemulsions, lipid nanoparticles (LNPs), solid lipid nanoparticles (SLNs), and nanostructured lipid carriers (NLCs)^370^ (Figure 9). In most of these nanoparticle structures, surfactants play a key role in their colloidal stabilization in aqueous environments.^371–373^ Although surfactants are strictly speaking not always lipids, due to the structural similarities between some lipids and surfactant molecules, in this section, we will refer to surfactants with hydrophobic tails as being lipid-based.

**Figure 9:**
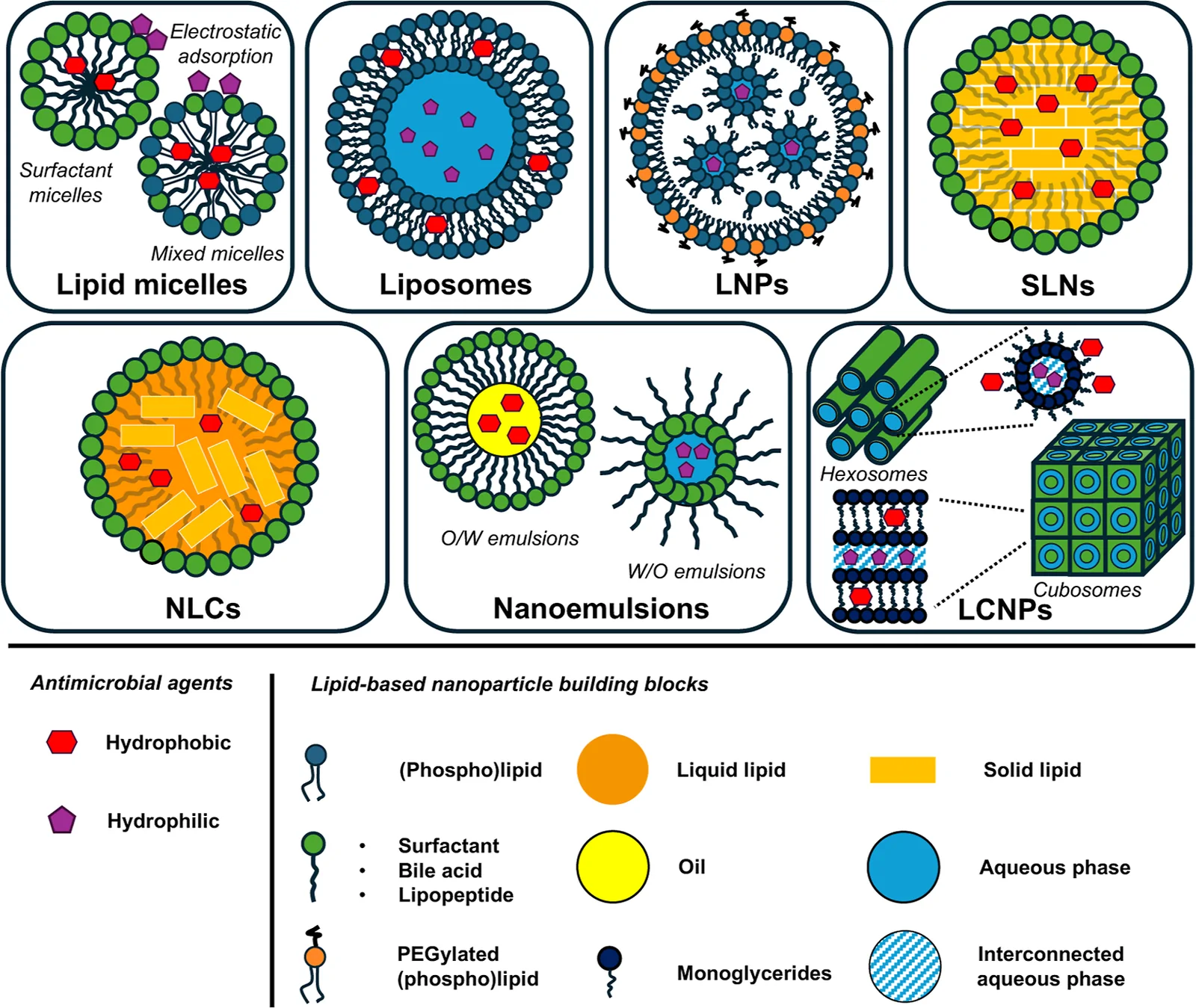
Overview of lipid-based antimicrobial delivery systems based on adsorption, encapsulation/entrapment, and supramolecular assembly. These include lipid micelles, liposomes, lipid nanoparticles, solid lipid nanoparticles, nanostructured lipid carriers, nanoemulsions, and liquid crystal nanoparticles. Lipid micelles are formed based on the self-assembly of surfactants, phospholipids, bile acids, and/or lipopeptides, allowing them to entrap antimicrobial agents, as well as electrostatically adsorb antimicrobial agents through the incorporation of charged compounds. Liposomes, consisting of a phospholipid double bilayer, resulting in a self-assembled vesicle shape, can encapsulate hydrophilic and/or hydrophobic antimicrobial agents. LNPs are typically composed of a combination of non-charged, charged, and PEGylated (polyethylene glycol/oxide (PEG/PEO)-conjugated) lipids, forming a particle with an interior based on lipid-mRNA or lipid-antimicrobial electrostatic complexation with associated aqueous domains. SLNs consist of a solid lipid matrix stabilized by a surfactant monolayer, resulting in a stable matrix for incorporating lipophilic antimicrobials. NLCs, a second-generation form of SLNs, combine solid and liquid lipids covered with a surfactant monolayer, allowing for the hydrophobic antimicrobial agent delivery. Nanoemulsions are kinetically stable dispersions of oil in an aqueous phase (o/w emulsion) or aqueous phase in an oil phase (w/o emulsion) that are stabilized by surfactants, allowing for the encapsulation of hydrophobic or hydrophilic antimicrobial agents in the oil or aqueous core. Finally, liquid crystal nanoparticles, including cubosomes and hexosomes, possess internal non-lamellar structures that allow the formation of highly interconnected aqueous phases in a lipid matrix, allowing for high drug loading and sustained release of both hydrophobic and hydrophilic antimicrobial agents.

Later, in the late 1990s, a new generation of lipid-based nanoparticles emerged, known as non-lamellar liquid crystalline nanoparticles (LCNPs), which include cubosomes and hexosomes^374^ (Figure 9). A major recent milestone in the history of lipid-based nanoparticles was the commercialization of LNP COVID-19 mRNA vaccines in 2020, resulting in the saving of millions of lives over the last five years.^375^ Lipid-based nanoparticles encompass a large versatility in structural characteristics and, therefore, can also facilitate the delivery of both hydrophilic and hydrophobic antimicrobials, depending on the platform used. Similar to polymer-based materials, lipid-based materials can be engineered as smart materials, where, after enzymatic cleavage, changes in pH or surface-charge-switching lipids can result in the release of the antimicrobial compounds.^376,377^ In the following subsection, we review recent advances in lipid-based approaches to antimicrobial delivery.

#### 5.3.1 Lipid-Based Micelles with Antimicrobial Load

Lipid-based micelles play an important role in drug delivery due to their ability to self-assemble and entrap therapeutic compounds. Similar to the previously discussed self-assembled lipopeptide micelles, such as those formed by daptomycin or surfactin (see Section 5.1),^260,261,378^ these systems rely on amphiphilic molecules with hydrophilic head groups and hydrophobic tails. During self-assembly, the hydrophobic tails orient toward the interior of the micelle, while the hydrophilic heads face the surrounding aqueous environment (Figure 9). In contrast to the systems described earlier, where the antimicrobial agents themselves form micelles, the focus here is on micelles that serve as carriers for antimicrobial drugs. When the micelle-forming compounds are uncharged, drug loading typically occurs through hydrophobic interactions, limiting entrapment to hydrophobic antimicrobials.^379^ However, if the micelle incorporates charged components, electrostatic interactions can facilitate adsorption of charged drug molecules, thereby broadening the range of deliverable agents.^380^

Beyond classical and biological surfactants (like synthetic compounds and bile acids,^381–383^ respectively), a variety of lipid-based amphiphilic molecules, including phospholipids^384^ and lipopeptides,^385,386^ form colloidally stable micelles capable of entrapping diverse antimicrobials. Additionally, a combination of these compounds can be used to prepare so-called mixed micelles, where two or more different amphiphilic compounds are used, offering additional flexibility and functionality in drug delivery systems.^387^ In the last decades, multiple different antimicrobial-loaded surfactant- and lipid-based micelles were investigated, including entrapment of antimicrobial agents like AMPs,^383,386,388^ macrolides,^380,381^ and β-lactams.^387^ Some surfactants and lipopeptides also exhibit intrinsic antimicrobial activity and can therefore serve both as micelle-forming components and antimicrobial agents.^385,386,389^ Overall, the field of surfactant- and lipid-based micelles for antimicrobial delivery remains relatively small. This is partly due to the limited number of hydrophobic antimicrobial agents suitable for entrapment in micelles, as well as the availability of more stable and versatile lipid-based nanoparticle systems, such as liposomes, solid lipid nanoparticles, and lipid–polymer hybrid nanoparticles, which often offer improved drug loading, stability, and controlled release.

#### 5.3.2 Liposomes

Liposomes, discovered in the 1960s, are spherical closed bilayer phospholipid systems with an aqueous core^368,390^ (Figure 9). They are typically composed of amphiphilic, charged, and/or “PEGylated” phospholipids, sometimes supplemented with cholesterol, that form a self-assembled vesicle through thin-film hydration methodologies.^32,390^ To decrease the size, lamellarity, and dispersity of the vesicles after hydration, methods such as sonication and extrusion can be used.^390^ The vesicle sizes of unilamellar liposomes are typically classified as small unilamellar vesicles (SUVs) (25–100 nm), large unilamellar vesicles (LUVs) (100–1000 nm), and giant unilamellar vesicles (GUVs) (>1 μm).^32^ Vesicles containing multiple concentric bilayers are referred to as multilamellar vesicles (MLVs), which, due to their multiple bilayer structure, can encapsulate higher quantities of drugs.^390,391^ They are, however, often less stable and less uniform, overall resulting in poorer drug delivery characteristics.^392^

Based on the contents, liposomal formulations can be divided into four categories: conventional liposomes, ligand-targeted liposomes (through conjugation of antibodies, peptides/proteins, or carbohydrates, depending on the desired target for delivery), sterically-stabilized liposomes (through use of PEGylated phospholipids), and combinations of these.^32,393^ For targeted delivery, liposomes can be engineered to be pH,^377,390^ heat,^32,394^ or light-responsive.^394^ Compared to other drug delivery systems, liposomes offer several advantages, including the ability to encapsulate both hydrophilic and hydrophobic drugs within the same structure, high biocompatibility, biodegradability, minimal toxicity, and usually have limited interaction with the immune system^390^ (Figure 9).

Shortly after their discovery in the 1960s, researchers began investigating liposomal formulations loaded with various antimicrobial agents.^243,395^ Research in this field rapidly expanded in the following years, focusing on the development of new liposomal formulations with various antimicrobial agents aimed at improving antimicrobial treatments against a wide range of bacteria and fungi.^243,396^ These advancements led to the first commercial antimicrobial liposomal formulation, where the antifungal agent amphotericin B (AmB) was encapsulated in liposomes to reduce its nephrotoxicity.^397,398^ The resulting liposomal AmB formulation, provided as a lyophilized powder, received its first clinical approval in 1989.^397^ Subsequently, in 1997, the formulation, marketed as AmBisome, received FDA approval in the US.^397^ In the 21st century, numerous antimicrobial-loaded liposomes have been developed, frequently showing improved therapeutic efficacy through enhanced antimicrobial activity, extended circulation times, and the capacity to overcome specific resistance mechanisms.^32^ In 2018, the second, and for now, the most recent, antimicrobial liposomal formulation was approved by pharmaceutical regulatory authorities.^399^ Liposome-loaded amikacin, encapsulated in approximately 300 nm liposomes, with the trade name Arikayce, was developed for pulmonary (nebulized) treatment of multidrug-resistant *Mycobacterium avium* complex (MAC) lung disease.^238,399,400^ The addition of Arikayce to standard therapy for MAC increased the treatment success rate from 9% to 29%.^399^

Despite the emergence of other, often more effective, lipid-based systems since the 1990s, research into antimicrobial liposomes has continued extensively.^243^ In recent decades, hundreds of formulations have been explored at the preclinical stage, incorporating diverse agents ranging from conventional antibiotics to antimicrobial monoclonal antibodies (mAbs),^401^ bacteriophages,^402^ AMPs,^239^ and metal-based nanoparticles.^396,403,404^ For detailed overviews of these investigated formulations, we refer to the following recent review articles: Ghosh & De (2023),^396^ Makhlouf et al. (2023),^403^ and Wang (2021).^404^ Although much less common, other vesicular surfactant- and lipid-based drug delivery systems have also been explored for antimicrobial delivery^405^ (Figure 10). Examples include exosomes, niosomes, transfersomes, and ethosomes. Exosomes are natural extracellular vesicles 40–200 nm secreted by cells involved in intercellular communication.^406^ They consist of a lipid bilayer and can, after collection, be loaded with antimicrobial compounds. Niosomes are vesicles composed of non-ionic surfactants and cholesterol that have relatively similar properties to liposomes, where both hydrophobic and hydrophilic antimicrobial compounds can be encapsulated.^407,408^ Although drug loading is comparable, niosomes often exhibit improved stability, cost-effectiveness, and ease of surface modification compared to liposomes.^407^ Antimicrobial transfersomes and ethosomes are both meant to target topical antimicrobial treatments.^405,409,410^ Transfersomes consist of phospholipids combined with edge activators (surfactants) that confer high deformability to enhance skin penetration,^411^ while ethosomes are vesicles made from phospholipids with high concentrations of ethanol, optimized for transdermal drug delivery by fluidizing skin lipids.^412^

**Figure 10:**
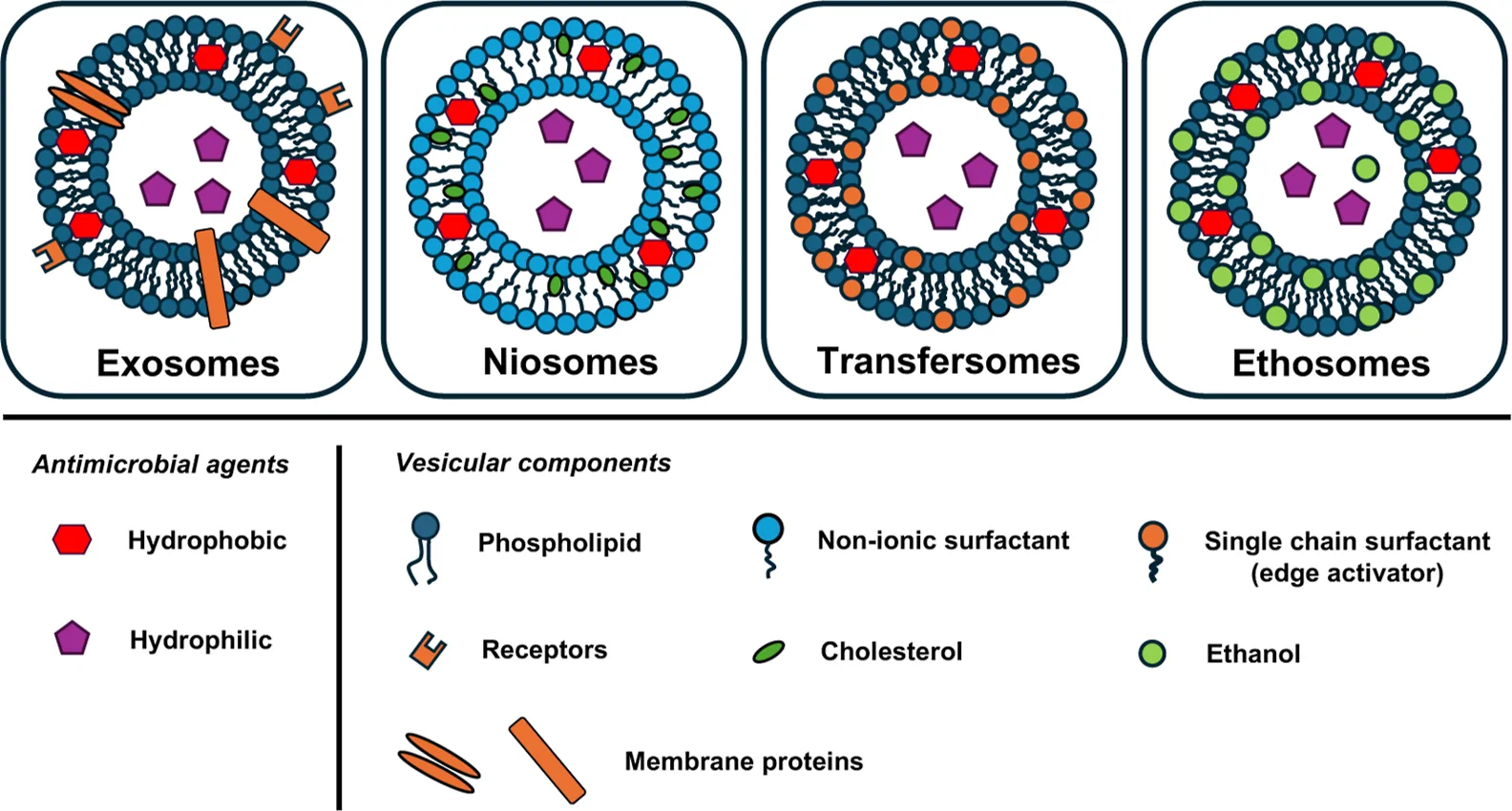
Illustrations of vesicular antimicrobial delivery systems of exosomes, niosomes, transfersomes, and ethosomes. These systems can host both hydrophobic and hydrophilic antimicrobial compounds. Phospholipids form the primary structural components of exosomes, transfersomes, and ethosomes. Exosomes are naturally occurring extracellular vesicles that also incorporate membrane proteins, receptors, and other biologically relevant molecules. Designed for topical delivery through skin lipid fluidization, transfersomes contain edge activators, typically single-chain surfactants, while ethosomes are characterized by their high ethanol content. Niosomes are vesicular entities formed from a combination of non-ionic surfactants and cholesterol.

Recent advances in antimicrobial vesicular and liposomal development have primarily focused on their interactions with bacterial membranes.^396,413^ Depending on their formulation, liposomes can fuse directly with bacterial membranes (fusogenic liposomes), adhere to the surface and subsequently break down to release antimicrobials, or be internalized by the bacteria through endocytosis, releasing their contents inside the cell. These mechanisms enhance local antimicrobial concentrations and can help bypass resistance mechanisms such as efflux pumps (Figure 2), facilitate drug uptake, and improve targeting of biofilms.^32,403^ To enhance fusion, adhesion, and endocytosis properties, liposomal formulations can contain cone-shaped lipids, such as DOPE (dioleoylphosphatidyl ethanolamine), as well as cationic phospholipids. DOPE facilitates membrane fusion by inducing negative curvature (increased liposome flexibility), promoting fusion.^32,414,415^ Cationic lipids enhance adhesion and endocytosis through strong electrostatic interactions with the negatively charged bacterial surface.^403,413^ While these interactions improve antibacterial efficacy, cationic liposomes are often associated with increased cytotoxicity toward mammalian cells.^403^ Currently, there is a novel liposomal formulation in clinical trials, CAL-02, that could play a role in bacterial infection management.^416^ CAL-02 is designed to treat severe pneumococcal pneumonia by neutralizing bacterial toxins rather than killing the bacteria directly. The formulation consists of engineered liposomes that act as membrane decoys that bind and sequester toxins released by pathogens. This prevents the toxins from interacting with host cells, thereby reducing tissue damage and inflammatory responses. In a Phase II randomized clinical trial involving patients with severe pneumonia, CAL-02 showed trends toward reduced organ dysfunction and inflammatory markers when supplemented to standard antibiotic therapy.

Despite widespread use of liposomes, successful commercialization, and significant drug delivery advantages, liposome formulations still face several challenges. Issues such as physical and chemical stability, limited drug solubility within the bilayer, leakage of encapsulated drugs, and potential degradation of phospholipids can compromise their effectiveness. Additionally, manufacturing scalability and reproducibility remain significant hurdles for consistent large-scale production. Overcoming these limitations will be crucial for unlocking the full clinical potential of liposomal drug delivery systems.^404^

#### 5.3.3 Lipid Nanoparticles

The term lipid nanoparticles is widely used in the literature to describe a range of lipid-based nanoparticles, including liposomes, SLNs, and NLCs.^417^ Following the successful development of COVID-19 mRNA vaccines in 2020, which prominently featured lipid nanoparticles as a delivery platform, the field underwent a paradigm shift. As these mRNA-loaded lipid nanoparticles gained significant attention, they evolved into a distinct category, now specifically referred to as lipid nanoparticles (LNPs). In this review, we will therefore use LNPs to refer to mRNA-LNPs-style nanoparticles, where ionizable lipids combined with neutral lipids facilitate the encapsulation of oppositely charged cargo through electrostatic interactions. mRNA-loaded lipid nanoparticles are commonly produced by rapidly combining an aqueous solution of charged cargo (most often the mRNA) with an ethanol-soluble lipid mixture. The resulting nanoparticles, typically of 80–150 nm, have an outer colloidally stable surface with an amorphous lipid-mRNA complex interior with aqueous pockets^418^ (Figure 9). LNPs usually include four main lipid components: ionizable lipids, zwitterionic phospholipids, cholesterol, and PEGylated lipids.^419^ The development of mRNA-LNPs already started in the late 1980s,^420^ but so far the research has predominantly focused on mRNA.^421^

Over the last decade, the first studies emerged in which LNPs were used as a tool to combat antibacterial infections and enable therapeutic delivery.^421–424^ These studies initially focused on RNA-based approaches, such as the use of mRNA encoding antimicrobial peptides^422^ and the delivery of CRISPR-Cas gene-editing systems.^423^ Moreover, Hou et al. (2020) incorporated cathepsin B-mRNA into vitamin C-LNPs aimed to treat multidrug-resistant bacterial sepsis,^422,424^ while Kim et al. (2024) loaded LNPs with Cas13a/gRNA expression vectors, a plasmid that was able to control bacterial infections, likely through activating the breakdown of ssRNA.^423^ The most recent addition to LNP drug delivery is the first report of a true antibiotic-LNP carrier.^421^ Ang et al. (2025) reported a polymyxin B-loaded LNP formulation, where the authors employed anionic lipid-mediated electrostatic encapsulation with the cationic polymyxin B, instead of the conventional cationic lipid-anionic mRNA encapsulation.^421^ Their optimized formulation demonstrated improved performance in cytocompatibility, *in vivo* tolerability, and pharmacokinetics, highlighting the therapeutic potential of LNPs for antimicrobial delivery. This study represents a significant advancement in lipid-based antimicrobials by extending the application of LNPs beyond nucleic acids to include antimicrobial peptides. As the field of antimicrobial LNPs remains in its early stages of development, the full therapeutic potential of these systems has yet to be realized. Future research will ultimately determine their viability in combating drug-resistant infections.

#### 5.3.4 Solid Lipid Nanoparticles

Solid lipid nanoparticles (SLNs) are lipid-based particles composed of a solid lipid core stabilized by surfactants, typically ranging in size from 50 to 1000 nm^425^ (Figure 9). Developed in the early 1990s as an alternative to liposomes, SLNs offer a biocompatible, stable, cost-effective, and scalable platform for the delivery of hydrophobic drugs.^426,427^ Although the delivery of hydrophobic drugs is more common and less challenging, SLNs can also be formed with double emulsion techniques, allowing for hydrophilic drugs to be incorporated in their structures.^428–431^ Common solid lipids used in SLNs include triglycerides, partial glycerides, waxes, and fatty acids, while surfactants such as poloxamers, lecithins, sodium cholate, phosphatidylcholines, and polysorbates are used to stabilize the dispersion.^425,432–434^ Additionally, SLNs exhibit a large surface modification potential for improving targeted drug delivery.^13,435^ General preparation methods include high-shear and high-pressure homogenization, ultrasonication, and solvent emulsification/evaporation techniques, allowing for obtaining high control over their particle size, dispersity, and drug encapsulation.^426^ SLNs provide controlled drug release due to the reduced mobility of drug molecules in the solid lipid matrix, and they can prolong drug retention at target sites.^432,433^ Despite these advantages, their ordered crystalline structure can limit drug-loading capacity and lead to expulsion of the payload during storage.^432^

Numerous preclinical studies on antimicrobial-loaded SLNs have been reported over the last decade, where SLNs were loaded with fluoroquinolones,^435,436^ AMPs,^428,437–439^ glycopeptides,^13,431^ among multiple other antimicrobial agents.^425,430^ These antimicrobial-SLNs are mostly meant for topical and parenteral administration,^426^ while some studies have focused on pulmonary delivery.^238,436,439^ Notably, SLNs have demonstrated the ability to penetrate bacterial biofilms and selectively accumulate at infection sites, enhancing targeted antimicrobial efficacy.^438,440^ Although SLNs have been extensively investigated for both topical and systemic antimicrobial delivery, significant challenges remain in enhancing drug entrapment efficiency and stability (inducing particle growth through Ostwald ripening), especially for hydrophilic compounds.^430,432^ Additionally, comprehensive studies examining the interactions between surfactants and drug payloads are still limited and require further investigation before any SLN formulation can enter clinical studies.^371^ While SLN sizes are generally controllable, they play a critical role in drug delivery performance and remain a notable limitation in many reported studies.^430^ Particle size can strongly influence biodistribution, with larger nanoparticles generally exhibiting greater uptake by the mononuclear phagocyte system and accumulation in organs such as the liver and spleen.^441^ To address some of these challenges, nanostructured lipid carriers (NLCs) were developed by partially substituting the solid lipid component with liquid lipids. This modification also improves drug loading capacity and enhances storage stability.^432^

#### 5.3.5 Nanostructured Lipid Carriers

Nanostructured lipid carriers (NLCs) represent the second generation of lipid nanoparticles, developed to address key limitations of SLNs.^442^ Like SLNs, NLCs are nanoparticles composed of biodegradable lipids that can both encapsulate hydrophilic and hydrophobic drugs (through double emulsion techniques), and they are typically produced using the same scalable methods such as high-pressure homogenization, ultrasonication, and solvent emulsification/evaporation.^425,442,443^ Both systems exhibit good biocompatibility, have surface modification potential, can be made in various sizes (50–1000 nm), and have similar routes of administration, including topical, oral, and parenteral delivery.^432,442,444^ SLNs and NLCs are structurally and methodologically very similar, and most of the differences solely stem from the absence and presence of liquid lipids in their formulations^442^ (Figure 9).

The incorporation of liquid lipids in NLCs is primarily responsible for their superior performance compared to SLNs in several critical aspects, including drug loading capacity, physical stability, and long-term retention of active compounds.^432,442,443^ NLCs are typically formulated by combining solid and liquid lipids in ratios where the solid lipid fraction still exceeds the liquid one, commonly starting from a 70:30 solid/liquid ratio.^443,444^ The lipids in NLCs often have relatively more unsaturated and/or shorter-chain fatty acids than in SLNs (*e.g.*, oleic acid (C18:1) instead of stearic acid (C18:0), and caprylic acid (C8:0) instead of palmitic acid (C16:0)). Modulating the quantity of liquid lipids in the formulation disrupts the crystalline order of the solid lipid matrix, resulting in structural imperfections that facilitate improved drug loading and retention.^443^ In contrast to SLNs, where drug molecules are mainly incorporated in molecular form within a highly ordered crystalline core, NLCs possess a more disordered or amorphous matrix. This provides additional space for accommodating drugs not only as individual molecules but also as amorphous clusters, thereby minimizing the likelihood of drug expulsion caused by polymorphic transitions during storage.

Similar to SLNs, NLCs are well suited for delivering antimicrobial agents, including those targeting biofilms and multidrug-resistant bacteria.^427,445,446^ During the last decade, a large number of preclinical studies have investigated the potential of antimicrobial-loaded NLCs, where, for example, fluoroquinolones,^446,447^ rifamycins,^448,449^ AMPs,^445,450^ and others,^451,452^ have been incorporated. Similar to other lipid-based nanoparticles, NLCs are still far from clinical application due to several significant technical and performance challenges, including high coagulation rates, lipid modification, phase separation within the lipid matrix, potential (long-term) toxicity, and poor loading efficiency for hydrophilic drugs.^453^ Therefore, future developments should focus on addressing cytotoxicity concerns and improving their formulations, for example, by optimizing surface properties, finding optimal lipid compositions, and hydrophilic loading capacity to enhance their drug delivery potential.^444^

#### 5.3.6 Nanoemulsions

Nanoemulsions and microemulsions have gained renewed attention in recent nanomedicine research due to their unique properties and potential for enhancing drug delivery.^454^ Microemulsions were first described around the 1950s as thermodynamically stable systems that form spontaneously due to ultra-low interfacial tension.^455^ They can self-assemble due to high surfactant concentrations (often >20%), and result in droplets that are between 10 and 100 nm in size. Nanoemulsions, on the other hand, have been developed more extensively in the 1980s and 1990s as kinetically stable colloidal dispersions, where immiscible liquids, either oil or water, are emulsified by surfactants into, unlike the name suggests, larger droplets, generally ranging from 20 to 500 nm^245,454,456,457^ (Figure 9). These emulsions can both overcome gravitational forces and resist viscosity-induced instabilities, enhancing their stability. To avoid confusion and maintain consistency throughout this review, we use the term nanoemulsions broadly to describe nanoscale emulsion droplets, regardless of their thermodynamic stability or formation (self- or driven-assembly).

These emulsions are formed as water-in-oil (w/o), oil-in-water (o/w) (Figure 9) or multiphase emulsions (*e.g.*, oil-in-water-in-oil (o/w/o) or water-in-oil-in-water (w/o/w)), allowing for a variety of drug loading possibilities, depending on the hydrophilicity of the drug in question.^245,458^ The oils are usually vegetable oil/seed oil-based and contain a mix of (medium chain) triglycerides, while surfactants such as Tween, poloxamers, and polyethylene glycol are commonly included, along with co-surfactants like glycerin and ethanol.^458^ Nanoemulsions have been shown to improve drug delivery properties by improving stability, solubility in aqueous media, and bioavailability.^459^ They also offer high biocompatibility and can be prepared using relatively simple techniques, making them an attractive option for pharmaceutical applications.^454^

Emulsions are usually prepared using high-pressure homogenization, microfluidization, and sonication. Additionally, there are some other, so-called “low-energy” approaches, such as temperature-induced phase inversion and spontaneous emulsification, *i.e.*, self-nanoemulsifying drug delivery systems (SNEDDS).^460,461^ SNEDDS are lipid-based formulations that spontaneously form nano-sized emulsion-dispersions in gastrointestinal fluids to enhance the oral bioavailability of poorly water-soluble drugs.^245,460^ The contents of SNEDDS usually involve a combination of surfactants and optimal water/isotropic oil combinations.^245,460,461^ Their emulsification is triggered by the mild agitation resulting from the movement and influx of gastrointestinal (GI) fluids. Upon entering the GI tract, SNEDDS form nanoemulsions that encapsulate the drug, thereby enhancing intestinal absorption.^245,460^ Although reports on antibiotic-loaded SNEDDS are still relatively recent and limited, they represent a promising strategy for antimicrobial delivery with substantial potential for future applications.^245,460–462^

In addition to oral delivery routes, nanoemulsions can be administered via multiple other routes, including pulmonary, nasal, transdermal, topical, and intravenous routes, from many different types of formulations, such as foams, liquids, gels, creams, sprays, and aerosols.^454^ Most antimicrobial nanoemulsions are designed to enhance the performance of poorly water-soluble antimicrobials by improving their efficacy, enabling organ- and cell-specific targeting, enhancing biofilm penetration, and addressing challenges related to antimicrobial resistance.^245,454,458^ Preclinical studies of the antimicrobial-loading of nanoemulsions have primarily focused on the delivery of poorly water-soluble (o/w emulsions) antimicrobial compounds,^245,454,459^ but also antibiotic w/o emulsions have been reported.^463^ Examples include the formulation of nanoemulsions loaded with fluoroquinolones,^460^ antifungal compounds,^464^ macrolides,^459^ oxazolidinones,^463^ and aminoglycosides,^465^ among many others.^454^ While preclinical studies on antimicrobial loading of nanoemulsions show potential for improving drug delivery and efficacy, most remain limited to *in vitro* experiments or early-stage animal models.^454^ For now, challenges such as stability, scalability, and biocompatibility hinder their progression toward clinical translation.

#### 5.3.7 Liquid Crystal Nanoparticles: Cubosomes and Hexosomes

Non-lamellar liquid crystalline nanoparticles (LCNPs) are nanostructured colloidal particles formed by the self-assembly of amphiphilic lipids in excess water.^466–469^ Their self-assembly results in the formation of internal nanostructures that resemble liquid crystalline phases, such as (inverse micellar) hexagonal (hexosomes) and (inverse) bicontinuous cubic mesophases (cubosomes)^468–470^ (Figure 9). Cubosomes most often display primitive (*Im*3*m*), double-diamond (*Pn*3*m*), or gyroid (*Ia*3*d*) symmetry, consisting of a single lipid bilayer that forms a continuous, highly ordered lattice interlaced with aqueous channels.^466,467^ Hexosomes are anisotropic structures characterized by cylindrical water channels organized in a hexagonal arrangement, each surrounded by a lipid monolayer.^466^ The discovery and first in-depth characterization of these liquid crystalline nanoparticles dates back to the 1980s, when their unique structural features and self-assembly behavior were first systematically explored.^471^ LCNPs are usually composed of amphiphilic lipids such as monoolein, phytantriol, or glycerol monooleate.^467,469,472^

Preparation of cubosomes and hexosomes typically starts with the formation of a viscous bulk phase by mixing lipids and stabilizers with water under heat, followed by dispersion into nanoparticles using high-shear techniques such as ultrasonication or microfluidization.^466,467^ Surfactants like poloxamers or Tween are commonly added to prevent aggregation and stabilize the dispersion,^473^ although due to some reported cytotoxicity issues, LCNPs without the use of stabilizers are being investigated.^474^ The final internal structure can be controlled and is typically dependent on formulation parameters, including lipid composition, temperature, water content, and ionic strength.^467^ Alternatively to bulk phase formation, so-called bottom-up methods can induce spontaneous cubosome or hexosome nanoparticle formation under controlled mixing conditions.^469,475^ The internal architecture of LCNPs allows simultaneous incorporation of hydrophilic drugs (in aqueous channels), hydrophobic drugs (within lipid bilayers), and even amphiphilic drugs (at the interfaces), supporting both versatility and sustained release.^476^ Hexosomes and cubosomes typically have external sizes ranging from 100 to 400 nm, with internal structural features between 5 and 50 nm.^466,469,477^ Drug loading can occur either during or after particle formation, depending on the properties of the drug and the preparation method.^472,478^ When ester-based lipids are used, enzymatic degradation by lipases can trigger antimicrobial release, which is undesirable in some contexts but exploitable as a controlled-release mechanism in therapeutic design.^479^ Other reported triggers for drug release include temperature, ultrasound, pH, and electrostatic changes.^467^

Overall, LCNPs are emerging as a highly promising platform for antimicrobial delivery, often aimed at topical, parenteral, or oral administration routes.^473,480^ During the last decade, preclinical research has focused largely on the incorporation of different antimicrobial agents in LCNP structures, such as aminoglycosides,^480,481^ AMPs,^474,478,481^ often human cathelicidin LL-37,^472,474,478^ macrolides,^476^ fluoroquinolones,^479^ glycopeptides,^476^ but also cocktails of antibiotics.^473,482^ Their capacity to eradicate biofilms was investigated,^480,481^ where significant improvements were found in the delivery of charged antibiotics via LCNPs.^481^ However, not all antimicrobial-loaded LCNPs are equally effective. While their protection from antibiotic degradation seems promising, some formulations see large reductions in their antimicrobial activity from the encapsulation in LCNPs.^472,478^ This highlights an important and essential capacity of drug delivery systems: the balance between therapeutic activity and adequate release.^472^

These systems require improvement and optimization of their therapeutic properties, dependent on the lipid composition, pore size, and release profiles, all dependent on the desired target, loaded antimicrobial, and administration route.^466–469^ In the search to improve these drug delivery platforms, LCNPs have been developed using polymer-based amphiphiles to form polymer-based hexosomes and cubosomes, offering greater structural versatility.^483^ However, as this is a relatively new field, no reports have yet been made of antimicrobial loading in these assemblies. Several key challenges must be addressed to advance the applications of LCNPs, including a deeper understanding of stabilizer–membrane interactions, effective tuning of pore sizes, comprehensive cytotoxicity studies, and enhancement of smart release capabilities.^467^

### 5.4 Hybridized Supramolecular Antimicrobial Drug Delivery Systems

Recent studies emphasize the potential of hybrid systems to address the limitations of traditional lipid nanoparticles and polymer-based nanoparticles, such as poor stability and rapid clearance.^484–486^ In many hybrid formulations, the role of the additional component is primarily supportive, for example by enhancing colloidal stability through PEGylation or providing a protective coating, rather than directly contributing to nanoparticle architecture or functionality.^484,487^

In this section, we define hybrid delivery systems as nanoplatforms composed of two or more distinct material classes, in which each component contributes to the structural, physicochemical, or biological properties of the nanoparticle. Consequently, formulations in which an additional material serves solely as a stabilizing or surface-modifying agent are not considered hybrid systems and will therefore not be discussed in the context of this review.

This field of science is rather diverse, where large numbers of different antimicrobial nanoparticles and material coatings are being developed in the preclinical stages of research. To maintain the structure of this review, the discussion will focus on the most extensively studied hybrid systems, illustrated in Figure 11.

**Figure 11:**
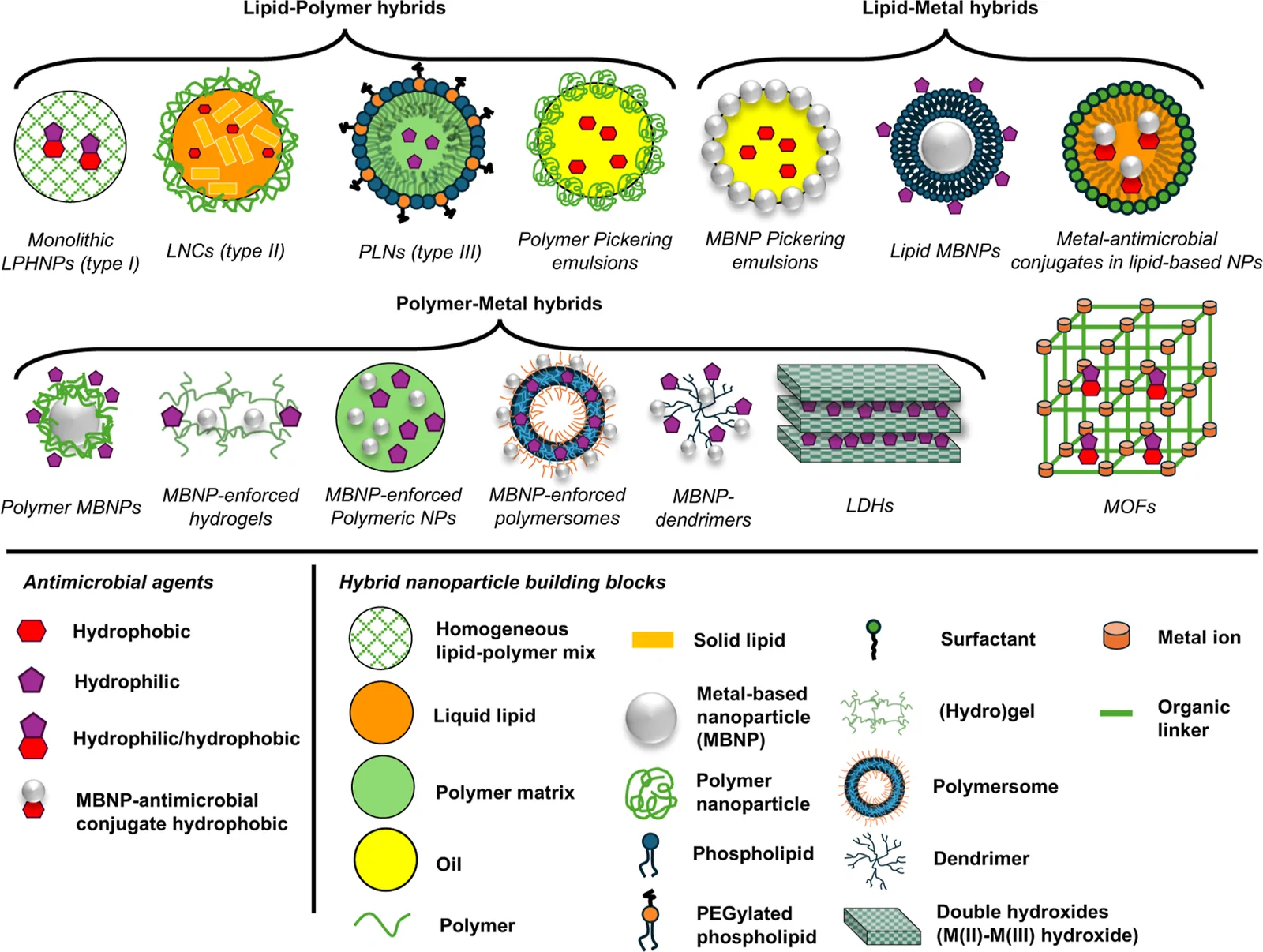
Illustrations of the most extensively studied hybrid nanoparticles for antimicrobial delivery, separated by classes of lipid-polymer hybrids, lipid-metal hybrids, polymer-metal hybrids, and metal-organic frameworks. Illustrated examples of lipid-polymer hybrids include monolithic lipid-polymer hybrid nanoparticles, lipid-core nanocapsules, polymeric lipid hybrid nanoparticles, and Pickering emulsions. Lipid-metal hybrids most often include metal-based nanoparticles, and examples excluded here are MBNP-Pickering emulsions, lipid-coated MBNPs, and antimicrobial metal-antimicrobial conjugates in lipid-based NPs. Polymer-metal hybrid examples include polymer-coated MBNPs, MBNPs-enforced hydrogels, polymeric NPs and polymersomes, MBNP-dendrimers and layered double hydroxides.

#### 5.4.1 Lipid-Polymer Hybrid Nanoparticles

By far the most studied hybridized supramolecular drug delivery systems are based on the use of a combination of polymers and lipids.^485,486^ Main types, relevant for antimicrobial delivery, include monolithic lipid-polymer hybrid nanoparticles (LPHNPs), lipid-core nanocapsules (LNCs), polymeric lipid hybrid nanoparticles (PLNs), Pickering emulsions, mixed lipid-polymer micelles/vesicles, liposomal hydrogels, dendrimer-loaded SLNs, and cyclodextrin-loaded liposomes.^243,485,486,488–490^ While the latter four systems share structural and functional similarities with conventional polymer- and lipid-based nanoparticles, and in some cases demonstrate superior antimicrobial delivery, they will not be discussed in further detail here. In these systems, polymers or lipids are combined/loaded with/in lipid-based or polymer-based nanoparticle solutions^488,491–494^ (see Figures 8 and 9).

In this subsection, we shift the focus to monolithic LPHNPs, LNCs, PLNs, and Pickering emulsions, which are systems that are structurally distinct from the nanoparticles discussed previously. While monolithic LPHNPs, LNCs, and PLNs are often collectively referred to as lipid-polymer hybrid nanoparticles, they will be categorized as Type I, II, and III, respectively.^485,486,495–497^ This classification is based on their structural differences, where Type I features a homogeneous matrix without a core–shell structure, Type II comprises a lipid-drug core surrounded by a polymer shell, and Type III a polymer-drug core that is covered by a lipid shell (Figure 11).

Monolithic LPHNPs (I), also known as mixed LPHNPs, are nanoparticles where the lipids and polymers are homogeneously distributed, resulting in stabilization of their structures and their load.^485,486^ They show structural similarities to PNPs, with the incorporation of solid lipids as in SLNs.^486^ They can be prepared using driven assembly techniques, such as nanoprecipitation, hot homogenization, and emulsification–solvent evaporation.^486,498,499^ The coexistence of lipids and polymers within a single uniform matrix often allows for the incorporation of hydrophobic loads within a polymer matrix. Additionally, with appropriate formulation design, hydrophilic loads can be encapsulated.^498,499^ LNCs (II) are structurally similar to NLCs (liquid/semi-solid) core, but instead of surfactants, the lipid cores are stabilized by polymers, which are often biodegradable and biocompatible polymers, such as PLGA or chitosan.^489,500^ LNCs can be prepared through deposition techniques, as well as emulsion-solvent evaporation and nanoprecipitation.^489^ Their core–shell architecture allows for high encapsulation efficiency of hydrophobic drugs, making LNCs effective by protecting unstable compounds and enabling targeted delivery, potentially enhanced further through ligand-functionalization of the polymeric shell. PLNs (III) consist of a polymer core with a lipid coating (shell) that combines the properties of both liposomes and PNPs into one compound, while simultaneously solving their disadvantages for delivery, such as low retention, early release, and low structural stability.^485,501^ A polymer, mixed with hydrophilic drugs, is loaded in the core, which is coated with a lipid single layer or bilayer, including PEGylated lipids for stability.^485^ Techniques to form PLNs include nanoprecipitation, emulsification–solvent evaporation, and sonication.^499,502^ PLNs are the most commonly explored lipid-polymer hybrid nanoparticle system within drug delivery as well as for antimicrobial delivery strategies.

Over the past decade, numerous preclinical studies have explored the use of lipid-polymer hybrid nanoparticles for antimicrobial delivery.^492,502–507^ Monolithic LPHNPs (I) were, for example, used as a drug delivery platform for a fluoroquinolone, norfloxacin,^503^ whereas (double-layered, polymer-lipid-polymer) LNCs (II) have been developed for the delivery of fusidic acid,^507^ and fluoroquinolones.^492^ Most papers in this field, however, investigated the properties of antimicrobial-loaded PLNs (III), most often with a poly­(lactic-*co*-glycolic acid) (PLGA) core and a cationic lipid coating.^502,504–506^ This was, for example, aimed at the delivery of fluoroquinolones,^502^ β-lactams,^504,505^ glycopeptides,^506^ and cycloserines,^504^ among others.^504^ While the understanding of the behavior and properties of Types I, II, and III lipid–polymer hybrid nanoparticles remains relatively limited, all three types demonstrate significant potential. Drawing from insights into structurally related systems, such as SLNs/NLCs, PNPs, and liposomes, these hybrids emerge as a promising strategy for advanced drug delivery applications. By combining the advantages of both lipids and polymers, controlled release, biocompatibility, and tunability are significantly improved. Nevertheless, systematic investigations into their structure–function relationships, long-term stability in physiological environments, and *in vivo* performance remain scarce, particularly in the context of infectious disease.

Pickering emulsions are o/w or w/o (nano)­emulsions that are emulsified similarly to the nanoemulsions discussed in Section 5.3.6, but instead of surfactants, are stabilized and modified through the adsorption of nanoparticles on the droplet surface^508–510^ (Figure 11). Their role in antimicrobial delivery is currently limited, with only a handful of reported examples, most of which focus on food safety, food packaging, antimicrobial coatings, and topical applications.^510–513^ Various stabilizing polymeric nanoparticles based on chitosan,^511^ starch,^512^ protein,^510,514,515^ and cellulose^516^ have been employed to formulate Pickering emulsions for the delivery of antimicrobial peptides^510,511^ and a range of other antimicrobial agents,^512,513,517^ including essential oils.^510,514,516^

Metal-based nanoparticles have also been employed as Pickering stabilizers, serving a dual role as both antimicrobial agents and emulsion stabilizers, thereby enabling the development of lipid-metal hybrid nanoparticles, for which other examples are discussed in Section 5.4.2).^518–520^ For example, Cai et al. (2016) developed electrospun Pickering emulsion nanofibers composed of poly­(lactic acid) (PLA) for tissue engineering applications.^518^ These nanofibers were loaded with vancomycin and stabilized using oleic acid and iron oxide nanoparticles. While this remains a specialized example, it highlights the interest of lipid-metal Pickering emulsions for the development of antimicrobial coatings, films, and tissue engineering strategies.

The use of Pickering emulsions for systemic antimicrobial delivery remains challenging, primarily due to the relatively large size of the emulsified droplets, which often fall in the micron rather than the nanoscale range.^511,515^ Consequently, their greatest potential currently lies in topical and coating antimicrobial applications, where droplet size is less restrictive.^512,516,517^ However, due to limited knowledge of structure–function-delivery relationships, further (pre)­clinical research is required to advance their development for effective antimicrobial delivery.^508^

#### 5.4.2 Lipid-Metal Hybrid Nanoparticles

Other types of lipid-metal hybrid nanoparticles purely aimed at antimicrobial delivery are relatively sparse. Although this field is developing, the focus of applications mostly goes in the direction of cellular labelling, bioimaging, and biocatalysis.^521^ Moreover, metal–lipid hybrids are typically designed to improve the stability and functionality of lipid-based nanoparticles, rather than to provide a direct antimicrobial effect systemically. For instance, incorporating metal nanoparticles onto the surface of liposomes or lipid assemblies can enhance stability and allow for stimuli-responsive release, triggered by factors such as pH, ionic strength, light, enzymes, or toxins.^521,522^ For antimicrobial applications, only a limited number of genuinely hybridized nanoformulations have been reported in which both lipid and metal components contribute to the structure or functionality of the nanoplatform.^432,523^

These include liposomes loaded with antibiotics, such as glycopeptides^522^ or tetracycline,^524^ which are coated with (chitosan-)gold nanoparticles, where their release can be triggered by bacterial toxins or lipases.^522,524^ In the second example, the antifungal drug clotrimazole was complexed with silver and encapsulated into SLNs.^525^ The last nanoformulation includes lipid-coated metal nanoparticles (silver and gold). They have primarily been developed to improve nanoparticle stability, rather than to enhance antimicrobial efficacy. Moreover, by limiting the release of metal ions, key contributors to their antimicrobial activity, this approach is considered undesirable for antimicrobial applications.^432,526,527^ An exception is the study by Mu et al. (2016), in which phosphatidylcholine-coated gold nanoparticles were loaded with gentamicin.^528^ The authors observed a synergistic effect between the gold nanoparticles and the antibiotic in eradicating both planktonic and biofilm-residing bacteria.^523,528^ Although metal–lipid hybrid nanoparticles, designed to improve antimicrobial activity, are still far from clinical application, their greatest potential lies in enabling stimuli-responsive therapeutics. This is driven by the dual role of metals as both antimicrobial agents and stimuli-responsive triggers, though toxicity concerns must still be addressed.

#### 5.4.3 Polymer-Metal Hybrid Nanoparticles

Polymer-metal hybrid nanoparticles have attracted growing interest in antimicrobial delivery, though their applications have been more extensively explored in materials science.^529^ With their property-enhancing capabilities as well as an additional antimicrobial effect, metal nanoparticles show significant potential when combined with polymeric nanostructures. While polymer-based nanoparticles often face limitations in stability and retention, incorporating metal nanoparticles can improve these properties and enhance functionalities such as targeted release.^170,529,530^ The preparation methods of polymer-metal hybrid nanoparticles are very diverse, as traditional preparation techniques for polymer-based nanoparticles, such as emulsion-solvent evaporation, nanoprecipitation, and self-assembly, often result in poor dispersion of metal nanoparticles in their structure.^529,530^ Instead, other techniques have been applied, such as electrospinning, sol–gel methods, *in situ* polymerization, and electrochemical deposition.^531^

Although only a handful of studies have been published on this topic, polymer-metal nanoparticles for antimicrobial delivery can be divided into four main categories. The first category is the conjugation of metal nanoparticles with dendrimers for antimicrobial delivery^291^ (Figure 11). Moreover, conjugation of two antimicrobials (often a metal-based nanoparticle and an antibiotic) can be expected to reduce the chances of development of resistance mechanisms in pathogenic bacteria.^291^ For example, Jiang et al. (2021) reported that dual-conjugated PAMAM dendrimers with the glycopeptide vancomycin and AgNPs exhibited improved antibacterial activity and decreased resistance susceptibility compared to vancomycin alone.^291^ The second category is metal nanoparticle-enforced hydrogel structures for antimicrobial delivery with topical applications. Metal-based nanoparticles are then usually added to improve hydrogel properties and for synergistic antimicrobial activity.^532,533^ Lopez-Carrizales et al. (2020) investigated ampicillin- and AgNP-loaded chitosan hydrogels for prophylactic treatment, due to their broad antimicrobial spectrum and biocompatibility with eukaryotic cells, to prevent early biofilm formation.^532^ In another study, Bursy et al. (2023) reported carbopol-based hydrogels containing chloramphenicol carried by various nanoparticles (AuNPs and SiNPs).^533^ The authors found they could reduce the chloramphenicol concentration by a factor of 4 while maintaining bacteriostatic activity, enabling less invasive treatment. The third category within polymer-metal nanoparticles is based on nanoassembly and is primarily aimed at topical or systemic antimicrobial delivery. Similar to the polymeric nanoparticles discussed and depicted in Figure 8, nanoassembly can be used to improve the functionality and properties of nanoparticles through the use of polymer-metal hybrids/conjugates.^529^ Over the last decade, examples of metal-incorporated polymersomes,^534,535^ polymeric micelles,^536^ polymeric nanofibers,^537^ polymeric nanocages,^538^ and polymer- and antibiotic-coated metal nanoparticles have been reported.^539,540^ These systems have successfully incorporated antimicrobial loads such as fluoroquinolones,^535^ β-lactams,^534,540^ and antimicrobial peptides,^538,539^ leading to enhanced antimicrobial efficacy, stability, and retention.

Lastly, although they differ somewhat from conventional metal nanoparticles described in Section 4.1, intercalated polymer-metal nanoparticle structures, most notably layered double hydroxides (LDHs), also known as anionic clays, have demonstrated promising potential for antimicrobial applications.^541^ LDHs typically consist of positively charged layers of a divalent metal in which a trivalent metal is isomorphically substituted, balanced by interlayer anions^541,542^ (Figure 11). When functionalized with polymers, these layers can be combined into organic–inorganic nanohybrids, which have promising applications in antimicrobial materials aimed at coatings, wound dressings, and tissue engineering.^243,543^ Examples have been reported of calcium–aluminum,^544^ magnesium–aluminum,^542,545^ and zinc–aluminum LDHs,^542^ where antibiotics such as ciprofloxacin,^544^ ampicillin,^542^ and cephalosporins^542,545^ have been successfully loaded with varying degrees of effectiveness. Although polymer-metal antimicrobial nanoparticles are a recent development, metal-based nanoparticles have already demonstrated the ability to significantly enhance the properties of polymer nanostructures. However, as for all antimicrobial delivery platforms with metal-based nanoparticles, concerns regarding their potential toxicity must be thoroughly addressed before these hybrid systems can be considered viable for therapeutic applications.

#### 5.4.4 Metal–Organic Frameworks

Metal–organic frameworks (MOFs) are porous crystalline materials made from metal ions or clusters linked to organic ligands via coordination bonds^546,547^ (Figure 11). They are considered a new generation of hybrid materials, combining inorganic and organic components into a single structure with drug delivery properties.^547^ The framework consists of metal nodes, which are often transition metals like iron, zinc, zirconium, or copper, connected to organic linkers such as carboxylates, phenolates, sulfonates, amines, phosphonates, or imidazolates.^548^ MOFs can encapsulate biomolecules during or after synthesis and are promising nanocarriers for drug delivery, biosensing, bioimaging, and biocatalysis.^546–548^ Drug loading strategies include pore encapsulation, direct assembly (cargo as ligands), and post-synthesis modification by surface linkage (covalent) or adsorption (non-covalent), although this is relatively restrictive due to their porosity characteristics.^549^ The cargo can be released based on stimulus responses, such as disease-specific triggers, as well as light, pH, and ionic strength changes. MOFs can be synthesized through various methods, including hydrothermal, solvothermal, microwave-assisted, and sonochemical techniques.^547^

The investigation of MOFs as antimicrobial materials and delivery systems has been extensively investigated over the past few decades.^547,548,550^ As well as antimicrobial loading in/on the MOFs, antimicrobial agents can also be used as a component of the MOF itself, as a linker, or as the metal ion.^547,548,551^ Their antimicrobial activity can be exerted through various mechanisms: releasing antimicrobial metal ions, releasing antimicrobial organic linkers, encapsulating and releasing active agents, or a combination of these strategies.^550,552^ While metal ion-based release is useful for topical use, the potential toxicity of silver and cobalt ions limits their suitability for systemic applications.^552^ In those cases, MOFs constructed from more biocompatible metals such as zinc and iron offer a safer alternative.^547,550,552^ Antimicrobial-based organic linkers, such as nalidixic acid, have also been explored,^551^ giving rise to so-called “BioMOFs”, a biocompatible alternative to conventional MOFs. MOFs generally show high loading capacities for drugs, but can be prone to destabilization caused by drug release.^550^ A wide range of MOF-based antimicrobial delivery systems has been extensively studied, including structures such as ZIF-8,^550,552,553^ UiO-66,^554^ and MIL-53/MIL-100.^555,556^ These were aimed at facilitating antimicrobial delivery for many different antimicrobial agents, such as fluoroquinolones,^554,557^ gentamicin,^558^ glycopeptides,^555^ β-lactams,^552,556^ sulfadiazine,^559^ as well as AMPs^553,560^ and metal-based nanoparticles.^550,559^ For a more comprehensive overview of these antimicrobial MOFs, we refer to the reviews written by Li et al. (2021),^550^ Livesey et al. (2023),^547^ and Kaur et al. (2020).^548^

### 5.5 Antimicrobial Composites for Coating/Material Applications

While the previous sections of this review have primarily focused on the delivery of conventional antibiotics and antimicrobial peptides, we briefly turn our attention here to developments in antimicrobial hybridized composite applications for antimicrobial coatings and materials. These systems, although not the primary focus of this review, represent an important and rapidly advancing area of research, particularly within the fields of biomedical coatings, with applications for wound dressings, implant devices, tissue engineering, among others.^25,170,561^ As previously discussed, multiple antimicrobial agents, such as antimicrobial polymers, metal-based nanoparticles, carbon-based nanoparticles, silicon-based nanoparticles, and hydrogels, have great potential as coatings. Biomedical coatings need to be durable, effective, and non-toxic in their application.^561,562^ To improve their properties or to incorporate antibiotics/AMPs and other antimicrobial agents, hybrid materials offer a versatile platform.^563,564^ By incorporating two or more components, the mechanical, but also the antimicrobial properties can be optimized, allowing for excellent durability of the materials along with broad-spectrum and long-lasting antimicrobial effects.^563,565^

Antimicrobial coatings can generally be classified as passive or active systems. Passive coatings prevent microbial adhesion through surface modifications, such as hydrophobic treatments and charge manipulation, thereby reducing the likelihood of biofilm formation.^566^ Active coatings, in contrast, release antimicrobial agents, such as metal ions, antimicrobial peptides, or antibiotics that directly inhibit/kill microorganisms upon contact.^565,567^ Although active coatings provide rapid antimicrobial effects, their performance may diminish over time due to the accumulation of bacterial debris, which can block active sites and reduce contact between the antimicrobial agent and target organisms.^565^ Key challenges include cost-effective synthesis, safety, mechanical durability, and long-term efficacy. To address these limitations, stimuli-responsive smart materials, which activate in response to pH shifts, temperature changes, or bacterial presence, provide precise, on-demand antimicrobial action, thereby boosting efficiency while reducing unnecessary exposure and toxicity.^170,519,563,564^

Developments of biomedical coatings are often aimed at orthopedic and dental implants, wound dressings, indwelling medical devices, tissue engineering scaffolds, and surgical instruments.^529,561,562^ Passive and active coating composites can be developed using Pickering emulsions,^516,519^ polymer-metal hybrids,^529,568^ or be based on silicon, clays, or carbon, and combinations thereof.^170,564^ Most of the investigated hybrid coatings focus on polymers with metals.^25,170,519,564,568^ For example, Wu et al. (2023) reported the formation of biocompatible and antimicrobial scaffolds composed of PLA and TEMPO-oxidized cellulose nanofibers stabilized by AgNP-based Pickering emulsions.^519^ Another important polymer for coating applications is chitosan, valued for its antimicrobial properties as well as its excellent functionality, biocompatibility, and versatility in both active and passive hybrid antimicrobial coatings.^529,563^ Other promising hybrids include carbon-based and metal­(oxide)-based hybrids.^569,570^ For example, carbon nanotubes have been conjugated with AgNPs to create materials with enhanced antimicrobial efficacy.^570^ Additionally, certain metal oxides, *e.g.*, titanium dioxide, act as smart materials by harnessing photocatalytic properties to create self-disinfecting surfaces through light-activated generation of reactive oxygen species.^565,566^

The field of (hybridized) antimicrobial coatings is rapidly advancing with engineered materials and multifunctional designs that enhance antimicrobial performance.^25,564^ These approaches offer improved control over coating behavior, enable targeted activation of antimicrobial effects, and help prevent biofouling and reduce AMR. Although the research is thorough and widely oriented, antimicrobial coatings still suffer from challenges such as rapid drug depletion, toxicity, chronic inflammation, and adverse immune response, which have to be addressed in the future before they can be commonly applied.^25^ For a more detailed overview of the developments within the field of antimicrobial composites for material applications, we refer to the reviews by Ren et al. (2022),^170^ Sahoo et al. (2022),^564^ and Ebenezer et al. (2025).^25^

## 6 Limitations and Future Perspectives

Antimicrobial compounds are essential for treating severe and life-threatening infections. Their clinical effectiveness is, however, increasingly undermined by bacterial resistance mechanisms. Multidrug-resistant bacterial strains are rapidly emerging and are currently developing faster than new antibiotics are being introduced. Consequently, there is an urgent need for the development of new and innovative antimicrobials, as well as for improving the efficacy of existing treatments. This can be achieved through advanced and intelligent drug delivery systems, enabling targeted delivery to infection sites, particularly those involving resistant pathogens. In 2024, the WHO published a policy brief on the antibacterial pipeline trends and recommendations, where the authors of this policy brief listed the main shortcomings of the current clinical pipeline.^571^ Criticism included the limited focus on multidrug-resistant Gram-negative bacteria, insufficient incentives for large enterprises, a general lack of innovation, a low number of new oral antimicrobial formulations, a shortage of new pediatric treatments, disparities in innovation and treatment needs between low- and high-income countries, and inadequate support for developing non-traditional antimicrobial agents. Based on these shortcomings, the WHO gave several recommendations, such as ensuring adequate resources (including push and pull incentives) and better stimulation of innovation and the development of non-traditional antimicrobial agents in small and medium-sized enterprises.

Antimicrobial nanoparticles represent a strategy that is highly relevant to these recommendations, as they offer opportunities to develop innovative therapeutic approaches beyond conventional antibiotics. Successful translation of these platforms could help address current gaps in the antimicrobial pipeline by enabling improved delivery, enhanced efficacy, and potentially new mechanisms of action less susceptible to existing resistance pathways. In the following subsections, we will discuss the current limitations of antimicrobial nanoparticles, including safety concerns, limited understanding of long-term biological effects, the potential emergence of nanomaterial and nanoparticle resistance, and barriers associated with clinical translation. Finally, we will provide future perspectives on how emerging technologies, including artificial intelligence, may support the development and translation of next-generation antimicrobial therapies.

### 6.1 Safety Concerns and Resistance to Antimicrobial Nanomaterials and Nanoparticles

Although antimicrobial nanoparticles offer promising alternatives to conventional therapies, their clinical translation requires a comprehensive understanding of their safety profile. Following systemic administration, nanoparticles are subject to interactions with proteins and recognition by immune cells, which can significantly affect their distribution throughout the body. Their physicochemical properties, including size, morphology, surface charge, and composition, determine circulation time, cellular uptake, tissue penetration, and clearance pathways.^20,188^ In particular, nanoparticles may accumulate in organs involved in filtration and the immune response, including the kidneys, mononuclear phagocyte system, liver, and spleen.^20^ Although clearance is highly influenced by nanoparticle-specific physicochemical properties, nanoparticles smaller than ≈ 10 nm generally tend to undergo renal clearance, whereas larger particles (typically above ≈ 100–150 nm) are more likely to be sequestered by phagocytic cells and subsequently cleared by the liver and spleen.^20,188,572,573^

Additionally, long-term exposure to non-biodegradable nanoparticles may increase the risk of chronic toxicity, due to prolonged tissue retention, persistent immune activation, oxidative stress, or interference with normal cellular functions.^574^ Therefore, the use of biodegradable or biocompatible carriers is key in antimicrobial nanoparticle development, as they can be cleared through established physiological pathways. Conversely, surface modifications such as PEGylation or biomimetic coatings may initially reduce immune recognition and prolong circulation time but can introduce long-term challenges, including the formation of anti-PEG antibodies and altered immune responses.^575^ Consequently, immunotoxicity should be evaluated alongside antimicrobial efficacy throughout preclinical development. Furthermore, characterization under biologically relevant conditions is essential, as protein-corona formation and other biomolecular interactions can alter nanoparticle behavior. Standardized preclinical evaluation protocols should therefore incorporate such conditions to enable reliable comparison between different antimicrobial nanoparticle platforms and facilitate their translation toward clinical applications. Such evaluations should include systematic assessment of physicochemical properties, pharmacokinetics, biodistribution, clearance, immunogenicity, endotoxin levels, acute and chronic toxicity, and therapeutic efficacy in relevant infection models.^574,575^

Lessons from approved nanoparticle-based cancer and vaccine therapies, such as liposomal, albumin-based, micellar nanoparticles (cancer), and LNPs (mRNA vaccines) can provide valuable guidance for establishing such evaluation frameworks, particularly regarding long-term safety assessment, biodistribution profiling and manufacturing consistency.^576,577^ Adapting these established approaches to antimicrobial nanoparticles may accelerate their development while ensuring rigorous assessment of safety and clinical feasibility.

Although antimicrobial nanoparticles are often considered less susceptible to conventional resistance mechanisms due to their multi-target modes of antimicrobial action, increasing evidence indicates that microorganisms can adapt to antimicrobial stress induced by nanoparticle platforms.^578^ Unlike conventional antibiotics, where resistance commonly arises through target modification, enzymatic degradation, reduced permeability, or active efflux (Figure 2), resistance or tolerance to nanoparticles may involve broader physiological adaptations. Repeated sublethal exposure may impose evolutionary pressure that selects for bacterial populations with increased tolerance through alterations in cell envelope composition, membrane permeability, enhanced EPS production associated with biofilm formation, metal binding mechanisms, and activation of cellular stress-response pathways.^578,579^

Metal-based nanomaterials represent an important example where resistance evolution requires consideration.^578,580^ Although metallic nanoparticles and released metal ions target multiple cellular processes, including membrane integrity, protein function, and oxidative homeostasis, prolonged exposure to sublethal concentrations may induce adaptive responses that reduce susceptibility.^580^ These include increased metal-binding capacity, altered metal uptake, efflux activation, and changes in stress-response pathways. Potential cross-resistance between nanomaterial exposure and existing antimicrobial resistance mechanisms should be further investigated, as adaptations to metal nanoparticles and metal ions may influence bacterial susceptibility to conventional antibiotics.^581,582^ For example, Lu et al. (2020) found that AgNPs and ionic silver facilitate the HGT of plasmid-borne antibiotic resistance genes at sub-lethal concentrations.^581^

For other antimicrobial nanoparticle platforms, resistance development remains less understood and is likely dependent on the specific physicochemical properties and antimicrobial mechanisms involved.^578,583^ The mechanism can be referred to as adaptive tolerance, and can result in alterations in membrane composition, reduced nanoparticle association, increased biofilm formation, and activation of general stress-response pathways. For antimicrobial delivery systems, resistance may also arise primarily from adaptation to the encapsulated antimicrobial agent rather than the nanoparticle itself. Therefore, systematic evaluation of long-term bacterial adaptation will be important to determine the clinical relevance of resistance development across different nanoparticle platforms. Overall, standardized evolutionary studies should be incorporated into nanomaterial and antimicrobial delivery development pipelines. Serial-passage assays under controlled exposure conditions can monitor changes in antimicrobial susceptibility over multiple generations, while genomic and transcriptomic analyses can identify underlying adaptive mechanisms. Furthermore, HGT studies, particularly for metal-containing nanomaterials, are important to assess potential broader evolutionary consequences of nanomaterial usage.^578,580^

### 6.2 Translational Barriers

Despite the substantial progress in the development of antimicrobial nanocarriers, successful translation from laboratory-scale discoveries to clinically available products remains challenging. A major barrier is the scalability and reproducibility of nanoparticle production.^584^ Many nanoparticle systems are initially developed using small-scale batch methods, which may not directly translate to industrial manufacturing. Large-scale production requires reproducible and robust processes capable of maintaining critical quality attributes, including particle size distribution, morphology, surface properties, drug loading, encapsulation efficiency, and biological activity. Implementation of scalable manufacturing approaches, together with process analytical technologies and quality-by-design (QbD) principles, will be essential to ensure consistent product performance.

The regulatory evaluation of complex antimicrobial nanoparticles is also challenging.^46,585^ Unlike conventional small-molecule antibiotics, nanoparticle-based therapeutics contain multiple interacting components, and their biological activity depends not only on chemical composition but also on physicochemical properties such as size, charge, structure, and functionality. Consequently, regulatory pathways require comprehensive characterization strategies and standardized analytical methods to define product composition, purity, potency, stability, and safety. Additionally, storage stability and shelf-life need to be well-known, particularly for advanced nanoparticle systems containing labile antimicrobial agents, biological components, or complex supramolecular structures.

Complex manufacturing, characterization, and quality control requirements may increase production costs and limit commercial feasibility compared to conventional antibiotics. Furthermore, short treatment durations and antimicrobial stewardship policies, *i.e.*, responsive antibiotic use, may reduce market incentives despite the significant clinical need. Moreover, early engagement with regulatory bodies will be essential to establish clear regulatory pathways, reduce uncertainty, improve predictability in development costs, and accelerate clinical translation. Standardized characterization frameworks, QbD-driven manufacturing strategies, regulatory engagement, scalability, and economic feasibility will be critical to bridge the gap between innovative nanotechnology platforms and clinically accessible antimicrobial therapies.^584,585^

### 6.3 Translational Readiness

To summarize the developments of different antimicrobial delivery platforms, we provide a summary table with some key parameters of investigated antimicrobial delivery-enabling systems and nanoparticle strategies, such as common particle sizes and administration routes. In the last column, we have added a translational readiness framework (TRL), qualitatively summarizing the relative stage of development of each antimicrobial drug delivery and nanoparticle platform on a scale from 1 to 9. The scale reflects the gradual progression from fundamental laboratory research and initial *in vitro* validation (TRL 1–3), through preclinical *in vivo* evaluation and formulation optimization (TRL 4–6), to advanced preclinical development and clinical evaluation or implementation (TRL 7–9) (Table 3). The assigned TRL ranges are qualitative estimates. As translational readiness is highly formulation- and application-dependent, an advanced TRL achieved by a specific formulation should only be regarded as indicative rather than universally applicable to all materials or formulations within that class.

**Table 3:** Summary Table of Antimicrobial Nanoparticle Platforms Discussed in This Review, Including Representative Typical Particle Sizes, Administration Routes (Parenteral: Intravenous and Intraperitoneal, Topical, Pulmonary, Oral, and Implant Coatings), and Translational Readiness Level Classification^b^

platform class	subtype/system	components/examples	typical sizes	administration routes	TRL (1–9)
metal-based and metal-dependent systems	metal-based nanoparticles	Ag, Au, ZnO, intermetallic NPs (*e.g.*, CuAg, AuAg)	10–100 nm	topical, implant	4–6
	metal salts	silver^a^, zinc, copper	molecular	topical	8–9
	antibiotic-siderophores	cefiderocol^a^ (Table 2) and siderophore–antibiotic conjugates	molecular	parenteral	8–9
	metal complexes	gallium complexes, bismuth compounds	molecular	topical, pulmonary, oral	3–5
carbon-based nanoparticles	carbon-based nanomaterials	graphene oxide, carbon dots, carbon nanotubes	2–200 nm	topical, pulmonary, implant	2–5
silicon-based nanoparticles	silicon-based nanoparticles	mesoporous silica nanoparticles, nanoscale zeolites	50–300 nm (pore diameters: 1–50 nm)	parenteral (MSN only), topical, implant	1–4
self-assembling antimicrobials	self-assembling antimicrobial peptides (AMPs) and analogues	cationic AMPs, peptoids, and polymer assemblies	5–150 nm	parenteral, topical, pulmonary	2–4
	antibiotic conjugates	biomolecule and metal conjugation	10–200 nm	parenteral, topical, pulmonary	3–5
polymer-based antimicrobials	dendrimers	PAMAM and cationic antimicrobial dendrimers	3–20 nm	parenteral, topical	3–6
	polymeric nanoparticles	PLGA/PEG/chitosan-based polymeric nanospheres and nanocapsules	20–300 nm	parenteral, topical, pulmonary, oral	4–6
	polymeric micelles	PEG–PLA, PEG–PCL, and PEG–PLGA block copolymer micelles	10–100 nm	parenteral, oral	4–6
	polymersomes	PEG–PCL and PEG–PLGA block copolymer vesicles	50–300 nm	parenteral, topical, pulmonary	3–5
	polyelectrolyte complexes	alginate/chitosan complexes, peptide–polymer complexes, oppositely charged polymer complexes	50–300 nm	parenteral, topical, oral	3–5
	coacervates	peptide–polymer and polymer–polymer complex coacervates	20 −300 nm	parenteral, topical	2–4
	nanogels	crosslinked chitosan/alginate/hyaluronic acid/PEG/PLGA polymers	20–200 nm	parenteral, topical, pulmonary	3–5
	microgels	chitosan/alginate/PEG/peptide microgels	1–100 μm	parenteral, topical	3–5
	hydrogels	chitosan/alginate/PEG/peptide hydrogels	macroscopic (>100 μm)	parenteral, topical	5–7
lipid-based systems	lipid micelles	antimicrobial-loaded lipid-based micelles, antimicrobial surfactant micelles	10–50 nm	parenteral, oral	5–7
	liposomes	PEGylated liposomes, amphotericin B liposomal formulation^a^, amikacin liposomal formulation^a^	50–300 nm	parenteral, topical, pulmonary	8–9
	vesicles	exosomes, niosomes, transfersomes, ethosomes	50–200 nm	parenteral, topical, pulmonary	3–5
	lipid nanoparticles	ionizable-lipid nanoparticles and vaccine-inspired LNP systems	80–200 nm	parenteral, pulmonary	4–6
	solid lipid nanoparticles	antimicrobial lipid-core nanoparticles	50–300 nm	parenteral, topical, oral	4–6
	nanostructured lipid carriers	antimicrobial mixed solid/liquid lipid nanoparticles	80–300 nm	parenteral, topical, oral	4–6
	nanoemulsions	antimicrobial nanoemulsions	20–300 nm	topical, pulmonary, oral	5–7
	liquid crystalline nanoparticles	cubosomes, hexosomes	100–300 nm	parenteral, topical, oral	3–5
hybrid systems	lipid–polymer hybrids	lipid-coated PLGA nanoparticles, core–shell systems	50–250 nm	parenteral, topical, pulmonary	3–6
	lipid–metal hybrids	lipid-coated silver/gold nanoparticles	50–200 nm	topical, implant	1–3
	polymer–metal hybrids	polymer-stabilized metal (Ag, Au) nanoparticles	50–300 nm	topical, implant	1–3
	MOFs	antimicrobial ZIF-8, MIL-series metal–organic frameworks	50–300 nm	parenteral, topical, implant	1–5

a Approved antimicrobial product.

b The TRL values represent qualitative estimates of the platform-level translational maturity of nanoparticle systems for antimicrobial delivery, rather than the regulatory approval status of individual formulations. These classifications are based on an adapted platform-level TRL framework ranging from 1 (early-stage research) to 9 (clinical implementation).

Overall, the most advanced translational progress has been achieved in the lipid-based nanomedicine field, with two antimicrobial nanoparticle formulations currently approved: liposomal amphotericin B and amikacin liposomal inhalation suspension.^395,399,400^ Beyond lipid-based systems, additional clinically translated antimicrobial innovations and advanced formulations include cefiderocol, a siderophore–antibiotic conjugate, and silver-based antimicrobial coatings and wound-care products.^202^ These examples demonstrate the clinical feasibility of nanoparticle-based strategies for antimicrobial delivery.

Among the antimicrobial nanoplatforms discussed in this review (Table 3), lipid-based and polymeric delivery systems currently show the greatest potential for further clinical translation and are well-aligned with several priorities highlighted in the WHO 2024 policy brief.^571^ The technical characteristics of these nanoparticle platforms often allow for treatment via oral administration routes, enabling opportunities for improved pediatric formulations and dosing strategies, and supporting the targeted treatment of Gram-negative bacteria while aiming to reduce systemic toxicity. To facilitate translation, early alignment with regulatory bodies and a focus on well-characterized platforms will be essential to ensure that safety, quality, and efficacy requirements are addressed throughout development.

### 6.4 Artificial Intelligence and Machine Learning: Antimicrobial Compound Discovery and Nanoparticle Design

More innovations within antimicrobial development can potentially be stimulated by Artificial Intelligence (AI) and machine learning.^586^ Recently identified antibiotic candidates that are neither part of conventional antibiotic groups nor antimicrobial peptides, such as halicin^587^ and abaucin,^588^ were discovered through deep learning models trained to predict antimicrobial activity in chemical spaces.^586^ A review by Sun et al. (2026) focuses specifically on AMPs, summarizing how modern generative AI approaches are used to design AMPs with enhanced activity and reduced toxicity.^589^ Another recent review by Crysler & de la Fuente-Nunez (2026) surveys the shift from traditional cultivation-based antibiotic discovery to integrated digital platforms that combine advanced screening, omics-driven mining, and generative AI to systematically design and identify novel, durable antibacterials.^590^ Such approaches open up the possibility of identifying entirely new classes of antimicrobial agents that might not be intuitively discovered through traditional screening.

AI could also be integrated into the design of new antimicrobials within existing classes and into the incorporation of antimicrobial agents into delivery systems and materials.^591,592^ The development of effective antimicrobial nanoparticles involves understanding complex relationships between physicochemical parameters, biological interactions, and therapeutic outcomes. AI and machine learning approaches can possibly provide opportunities to identify structure–function relationships and thereby more effectively guide the development of nanoparticle characteristics, including size, shape, surface charge, composition, drug loading, and release behavior. By taking advantage of these models, antimicrobial nanoparticle formulations can be optimized for specific targets, predicting host–pathogen interactions, and allowing for the modeling of toxicity profiles before *in vivo* testing.

Furthermore, AI and machine learning approaches may contribute to understanding nanoparticle–bacteria interactions by predicting mechanisms of bacterial uptake, membrane disruption, biofilm penetration, and potential toxicity toward host cells. These approaches have recently been increasingly explored for the optimization of nanoparticle systems, including the design of ionizable lipids for LNPs, polymer-based delivery systems, and formulation parameters affecting nanoparticle performance.^591,593,594^ Beyond formulation design, AI and machine learning approaches may also support process optimization and scale-up possibilities, as demonstrated for ZnO nanoparticle production by Jose et al. (2021).^595^ However, broader implementation will require standardized datasets, improved experimental reproducibility, and careful validation of model predictions using biologically relevant systems.^591,592^ As antimicrobial nanoparticles move toward more complex and personalized therapeutic strategies, AI-guided design may become an important enabling technology for accelerating discovery and improving translational success.

To summarize, in this review, we have discussed a large variety of preclinical antimicrobial delivery systems, including both inorganic (metal-based, silicon-based) and organic systems, such as polymeric micelles, dendrimers, PNPs, vesicles, complexes, hydrogels, lipid micelles, liposomes, lipid nanoparticles, emulsions, and hybrid nanoparticles. Many of them have already demonstrated clear potential in improving the pharmacokinetics and targeted delivery of antimicrobial agents in preclinical settings. By improving drug solubility, circulation time, and tissue penetration, such systems can help revitalize existing antibiotics and facilitate the delivery of newer therapeutic candidates. However, as discussed in this review, these promising technologies still face considerable hurdles before widespread clinical implementation. Bridging the gap between laboratory innovation and clinical implementation remains the central challenge for antimicrobial nanomedicine.

Many types of nanoparticles, including metal-, carbon-, certain polymer-, and lipid-based systems, have been shown to cause cellular toxicity and induce immunogenicity in both laboratory and therapeutic settings.^170,172,596,597^ These adverse effects contribute to the complexity and unpredictability of regulatory approval. Antimicrobial development is costly and is therefore currently dependent on high-risk investments. Overcoming these barriers will require coordinated efforts from researchers, industry, and policymakers, as successful translation of antimicrobial delivery systems will also depend on supportive regulatory bodies and policy frameworks.

The field of antimicrobial nanoparticles holds great promise, but its future depends on addressing significant scientific, regulatory, and economic challenges in a responsible and sustainable manner. A cautious, evidence-based approach, including sustainable large-scale production and early integration of efficacy and toxicity assessments, will be critical to ensure that these therapies transition successfully from the laboratory to clinical settings. Furthermore, promoting the responsible and sustainable use of new antimicrobials and antimicrobial formulations will be essential to minimize unnecessary selective pressure, ensure that their long-term benefits outweigh potential risks, and ultimately expand our therapeutic arsenal against resistant infections.

## Vocabulary

Antimicrobial resistance: the ability of microorganisms to survive or continue growing despite exposure to antimicrobial agents that are intended to inhibit or eliminate them.
Antimicrobial nanoparticle: a nanoscale material, typically ranging from a few to several hundred nanometers in size, designed either to exhibit intrinsic antimicrobial activity or to function as a carrier for the delivery of antimicrobial compounds.
Soft matter: an interdisciplinary field of research focused on the study of complex materials whose structures and properties are governed by weak, reversible interactions. It encompasses systems such as polymers, lipids, colloids, and biomolecular assemblies, where molecular interactions drive dynamic organization, self-assembly, and responsiveness to environmental conditions.
Biofilm: a surface-associated community of microorganisms attached to a surface and embedded within a self-produced extracellular polymeric matrix that enhances protection against antimicrobial agents.
Supramolecular assembly: the organization of molecular components into larger functional structures through reversible, non-covalent interactions, including hydrogen bonding, van der Waals interactions, electrostatic interactions, hydrophobic effects, and π-π interactions.
Self-assembly: the spontaneous and reversible organization of molecules or nanoscale building blocks into ordered structures driven by intrinsic non-covalent interactions, without the need for external direction.
Clinical translation: the process of advancing a therapeutic strategy from laboratory research through preclinical evaluation and clinical development toward regulatory approval and clinical implementation.
